# Novel insights into negative pressure wound healing from an in situ porcine perspective

**DOI:** 10.1111/wrr.12971

**Published:** 2021-10-07

**Authors:** Jacob G. Hodge, Ashley L. Pistorio, Christopher A. Neal, Hongyan Dai, Jennifer G. Nelson‐Brantley, Molly E. Steed, Richard A. Korentager, David S. Zamierowski, Adam J. Mellott

**Affiliations:** ^1^ Bioengineering Graduate Program University of Kansas Lawrence Kansas USA; ^2^ Department of Plastic Surgery University of Nevada Las Vegas Las Vegas Nevada USA; ^3^ KIDDRC Imaging Core Facility University of Kansas Medical Center Kansas City Kansas USA; ^4^ Department of Pathology and Laboratory Medicine University of Kansas Medical Center Kansas City Kansas USA; ^5^ Department of Anatomy and Cell Biology University of Kansas Medical Center Kansas City Kansas USA; ^6^ Department of Pharmacy Practice University of Kansas Lawrence Kansas USA; ^7^ Department of Plastic Surgery University of Kansas Medical Center Kansas City Kansas USA

**Keywords:** biomedical engineering, foreign body response, genomics, negative pressure wound therapy, porcine model, wound healing

## Abstract

Negative pressure wound therapy (NPWT) is used clinically to promote tissue formation and wound closure. In this study, a porcine wound model was used to further investigate the mechanisms as to how NPWT modulates wound healing via utilization of a form of NPWT called the vacuum‐assisted closure. To observe the effect of NPWT more accurately, non‐NPWT control wounds containing GranuFoam™ dressings, without vacuum exposure, were utilized. In situ histological analysis revealed that NPWT enhanced plasma protein adsorption throughout the GranuFoam™, resulting in increased cellular colonization and tissue ingrowth. Gram staining revealed that NPWT decreased bacterial dissemination to adjacent tissue with greater bacterial localization within the GranuFoam™. Genomic analysis demonstrated the significant changes in gene expression across a number of genes between wounds treated with non‐NPWT and NPWT when compared against baseline tissue. However, minimal differences were noted between non‐NPWT and NPWT wounds, including no significant differences in expression of collagen, angiogenic, or key inflammatory genes. Similarly, significant increases in immune cell populations were observed from day 0 to day 9 for both non‐NPWT and NPWT wounds, though no differences were noted between non‐NPWT and NPWT wounds. Furthermore, histological analysis demonstrated the presence of a foreign body response (FBR), with giant cell formation and encapsulation of GranuFoam™ particles. The unique in situ histological evaluation and genomic comparison of non‐NPWT and NPWT wounds in this pilot study provided a never‐before‐shown perspective, offering novel insights into the physiological processes of NPWT and the potential role of a FBR in NPWT clinical outcomes.

**Abbreviations**
FBR: foreign body response
GAG: glycosaminoglycans
H&E: hematoxylin & eosin
HPF: high powered field
MMP: matrix metalloprotease
NBF: neutral buffered formalin
NPWT: negative pressure wound therapy
ROI: regions of interest
ROS: reactive oxygen species
VAC: vacuum‐assisted closure

## 1 INTRODUCTION

The term, ‘wound healing’, is a very broad way to describe a highly complex yet predictable set of cascading events that occur in the setting of tissue damage that has resulted from loss of tissue structure and function.^1^, ^2^ When our bodies incur a form of tissue damage resulting in either superficial, deep, or even structural damage, the skin is often left traumatized and exposed. The subsequent series of systematic events impartially affect most types of wounds and tissue after an injury. Those defined events are *haemostasis*, which results in tissue exposure to blood proteins, platelet activation, clot formation and provisional fibrin matrix formation.^3^ Next is *inflammation*, which is a series of inflammatory signals that results in immune cell migration into the wound site and removal of damaged debris and bacteria.^4^ This is followed by the *proliferative* phase, the stage of granulation tissue formation that includes neovascularization, fibroblasts proliferation and wound contraction.^5^, ^6^ Fibroblasts become the key contributor during this phase and begin dispersing throughout the wound site to prepare the tissue for the final stage of *remodelling*, which occurs after wound closure from reepithelialization.^7^ Fibroblasts delicately perform the remodelling process through a synchronized balance of collagen deposition and simultaneous degradation via secreted enzymatic factors, such as matrix metalloproteases (MMPs).^8^, ^9^ The overall purpose of this cascade of events is returning the tissue to a state of anatomical homeostasis and restoration of function.^2^ These four distinct stages are used to define our body's initial response to tissue damage, also known as acute wound healing.

The complex and dynamic nature of wound healing often can result in perturbation of acute wound healing, leading to pathological wound healing.^9^, ^10^ Pathological wound healing can be thought of as a continuum of physiologic healing where an aberrant process leads to an imbalance. One such imbalance can lead to excessive scar tissue formation and fibrosis.^11^, ^12^ Conversely, with insufficient scar tissue formation there is a deficit in healing which can result in ulcer formation.^11^, ^13^ Abnormally healing wounds can become chronic and result in complicated, non‐healing wounds accompanied by chronic inflammation.^11^, ^14^ There are several systemic and local factors that can have a negative influence on wound healing leading to chronic inflammation and non‐healing wounds, including the presence of an infection or foreign body.^15^, ^16^, ^17^

Infection within a wound site triggers a proinflammatory response that prompts recruitment of neutrophils, production of reactive oxygen species (ROS) and proteases, and subsequent tissue damage.^18^, ^19^ Inability to resolve the infection can result in chronic inflammation and a sustained state of non‐healing.^20^, ^21^ The foreign body response (FBR) is characterized by adsorption of plasma proteins onto a foreign object, which serves as both a biological stimulus and an anchor point for inflammatory cells.^22^, ^23^ A subsequent series of inflammatory signalling events results in a transition from acute to chronic inflammation, granulation tissue deposition, neovascularization and a phenotypic switch from M1 to M2 macrophages.^16^, ^24^ Following failed attempts of ‘frustrated’ macrophages to phagocytose the foreign object, foreign body giant cells (FBGC) are formed, which are multinucleated giant cells derived from fused macrophages that aid in the fibrotic encapsulation and/or expulsion of the foreign object from the body.^16^, ^24^ Failure to expel the foreign object from the body results in a sustained stimulus and chronic inflammation.

To this day, there is yet to be a singular type of wound care modality proven most effective for all wounds. However, occlusive or semi‐occlusive dressings that create and maintain a moist environment are considered the mainstay of wound care.^14^, ^15^ A recent and innovative strategy for wound care is the vacuum‐assisted closure (VAC) system. The VAC system is a form of negative pressure wound therapy (NPWT), which has gained increasing interest since its inception in 1997.^25^, ^26^ The VAC has shown clinical efficacy in a number of settings including surgical wounds and preparation of surgical wound sites for closure or grafting, traumatic wounds, skin grafting, complex ulcerative wounds (diabetic, pressure and venous stasis induced), and wounds involving exposed bone and orthopaedic implants.^27^, ^28^, ^29^, ^30^ The VAC system consists of inserting an open‐cell reticulated polyurethane‐derived foam dressing into a wound, called GranuFoam™, followed by sealing the wound site by applying a semi‐permeable adhesive film over the wound and foam dressing. The VAC system is then attached to a subatmospheric pressure system, typically set at 125 mmHg for this material. The open‐cell reticulated characteristic of the foam provides equal distribution of forces throughout the wound site and the ability for air and fluid to freely pass through the foam.^25^, ^31^ Application of subatmospheric pressure results in contraction of the reticulated foam and a mechanical decrease in wound site volume, thus bringing the wound edges closer together. This is similar to how sutures close surgical wounds or a compression bone plate bridges together two pieces of bone.^32^, ^33^ Moreover, exposure of wounds to the subatmospheric pressure VAC system has been claimed to enhance blood flow, remove excess fluid, decrease bacterial load, promote cellular proliferation, stimulate granulation tissue formation and expedite the overall wound healing process.^25^, ^26^, ^31^, ^34^

A current hypothesized mechanism of how the VAC system decreases bacterial load revolves around increased oxygenation from augmented neovascularization within the wound.^25^, ^31^ The increased circulation leads to improved neutrophil recruitment and the higher abundance of oxygen provides means for neutrophil oxidative burst activity.^35^ However, the data surrounding this mechanism are not fully understood and requires further study.^36^, ^37^ Clinically, bacterial burden is of high interest to physicians due to the negative impacts it can have on proper wound healing.^19^ Although bacteria are known to adhere to plastic implants or other devices, they have not yet specifically been shown adhering or proliferating directly on GranuFoam™. Yet, blood and matrix proteins, such as fibrinogen and vitronectin, are known to adsorb onto biomaterials upon implantation into the body.^23^, ^38^ Plasma protein adsorption results in modulation of the inflammatory response and subsequent cellular colonization and matrix deposition within the pores of the foam, known as ‘enmeshing’.^24^, ^39^ Therefore, the FBR can be thought of as a stimulus for tissue enmeshing due to the promotion of fibrous matrix deposition and encapsulation of the foreign body.^24^ Moreover, plasma protein‐coated plastics have been shown to enhance the adhesion and proliferation of bacteria.^38^, ^40^, ^41^ Thus, plasma protein adsorption and tissue enmeshing together could provide seed points for bacteria to adhere to and propagate within the GranuFoam™. Consequently, it is possible that there is a higher bacterial presence than originally thought, due to bacteria residing within the foam and the foam surface interacting with the wound environment.

**FIGURE 1:**
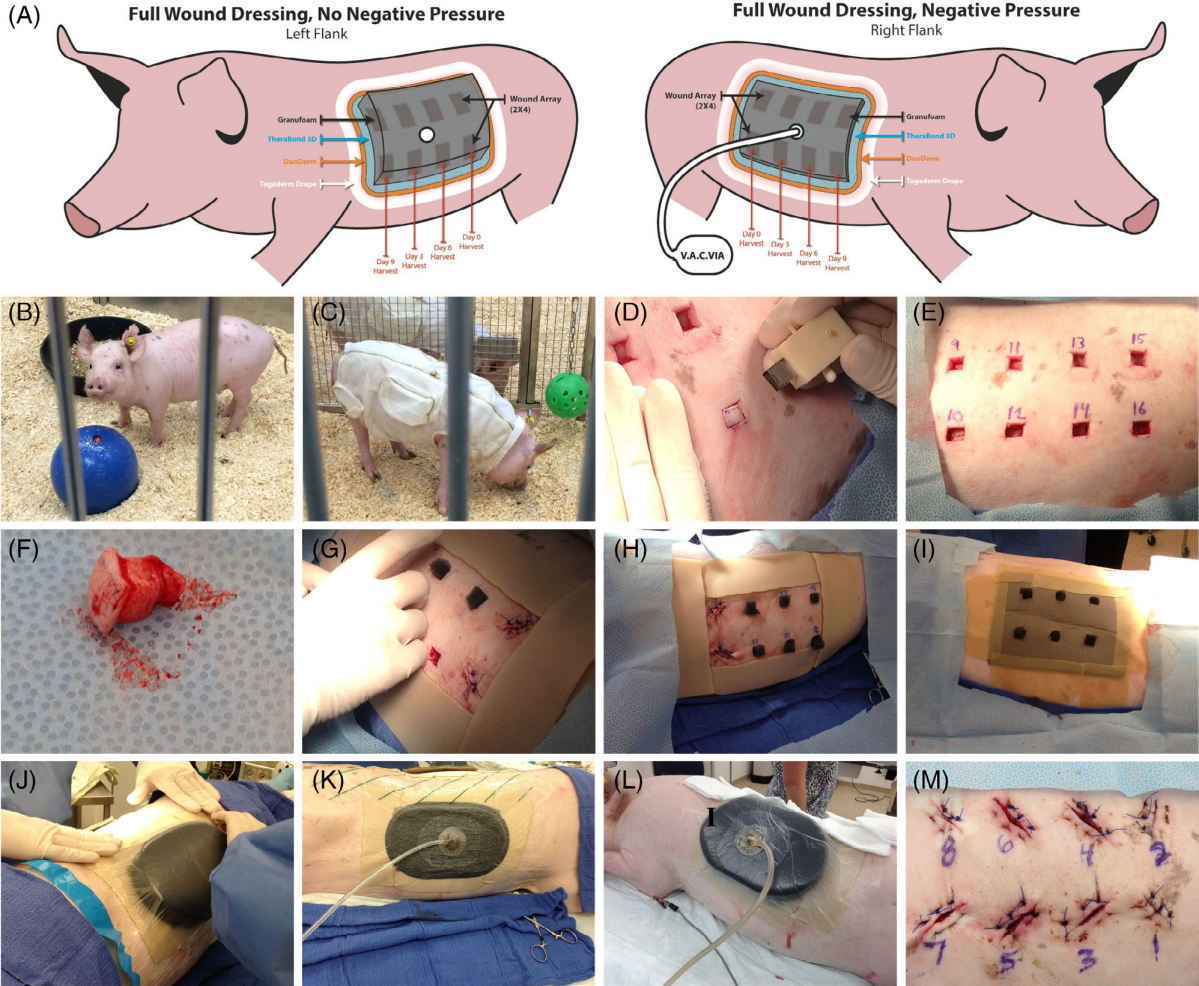
Surgical procedure overview. (A) Two arrays consisting of eight full‐thickness wounds (1 cm^3^) each were made on the back of two female Yucatan Miniature Pigs (*n* = 2) with a custom biopsy wound punch. The same dressing configurations were applied to both wound arrays. NPWT was applied using the KCI/Acelity VAC Via™ unit to the wound array on the right side, but not to the wound array on the left side of the animal. Dressings were changed, clinical images were taken and elliptical excisional explants were collected for tissue analysis of wounds at 0, 3, 6 and 9 days post‐surgery. (B) Animals were allowed to acclimate to the facility 2 weeks prior to surgeries. (C) Five days prior to surgeries, animals were fitted with custom protective jackets to house the vacuum pump and protect wound sites. (D) 1 cm^3^ full‐thickness wounds were made with a custom 3D‐printed biopsy punch. (E) A 2 × 4 wound array was made on each flank of each animal. (F) Biopsied tissue was cleanly removed and was preserved for histological and genetic analysis. (G) KCI/Acelity GranuFoam™ plugs (1 cm × 1 cm × 2 cm) were inserted into each wound. (H) The perimeter of each wound array was protected by DuoDerm® dressings. (I) A TheraBond® 3D Antimicrobial dressing was placed around the wound array with 1 cm^2^ openings pre‐cut to enable GranuFoam™ plugs to protrude. (J) A large GranuFoam™ Bridge was placed over the wound array to directly interface with the GranuFoam™ plugs. Afterward, a VAC semipermeable Tegaderm™‐like drape was securely placed over the wound array to make an airtight seal. A 1 cm^2^ hole was cut in the drape, and a vacuum port was attached. (K) Wound array under NPWT results in GranuFoam™ compression. (L) Wound array without NPWT (foam is not compressed). (M) Wounds sutured closed after elliptical excision of the wound with GranuFoam™ in situ for analysis

In this pilot study, a porcine wound array was developed to establish a holistic and temporal perspective for the evolution of the wound healing process and expand upon the original study performed by Morykwas and Argenta.^25^, ^26^ A porcine model was used due to the similarities between the wound healing processes of pigs and humans (Figure 1). The mechanisms behind how the VAC system exerts its effect on the wound healing process over time was assessed by controlling for the effects of the GranuFoam™ dressing without subatmospheric pressure. The impact of removal and reapplication of the foam dressing on wound healing was evaluated, in addition to how the VAC system may be mitigating bacterial load. We aimed to determine the relationship between the foam dressing and bacterial burden in situ, compared to the traditional method of ex vivo biopsy analysis of the wound bed without the dressing, which to our knowledge has never been investigated up to this point. We hypothesized that protein adsorption and tissue enmeshing within the GranuFoam™ is providing potential seed points for bacteria to adhere to within the GranuFoam™ dressing with increased protein adsorption and enmeshing due to exposure to subatmospheric pressure. Proliferating bacteria within the GranuFoam™ between dressing changes could be negatively augmenting the wound environment. This pilot study provides a new perspective to the mechanism of bacterial mitigation by the VAC system. Additionally, the non‐NPWT control group paired with the in situ perspective in this study provides novel insight into the potential role of a FBR to the GranuFoam™ dressing as a possible key component to outcomes seen in NPWT.

## 2 METHODS

### 2.1 Animals

Animal studies were approved by the University of Kansas Medical Center (KUMC) Institutional Animal Care and Use Committee (IACUC) under animal care and use protocol (ACUP) #2016‐2319. Two female 4.2‐month‐old miniature Yucatan pigs weighing 30–40 kg were procured from Sinclair Bio‐resources (Auxvasse, MO), and allowed to acclimate for 14 days in an AAALAC accredited facility at KUMC. Animals were provided with food, water and social enrichment ad libitum.

### 2.2 Surgeries, sample preparation and necropsy

Surgeries were performed sequentially on animals, with the same animal operated on in the morning while the other animal was operated on in the afternoon for all procedures. Animals were placed under general anaesthesia and ophthalmic lubricating ointment was placed to protect the eyes. The animals were prepped with three alternating scrubs of betadine and alcohol. A sterile surgical drape was placed over the animal and a hole to expose the surgical area was cut in the drape. A custom biopsy punch was used with a 3D‐printed acrylonitrile butadiene styrene stencil guide to create two rows of four full‐thickness wounds that were approximately 1 cm long by 1 cm wide by 1 cm deep on both the left and right side of the animal's back for a total of 16 wounds on each animal. Biopsies were bisected and preserved as baseline tissue controls in neutral buffered formalin (NBF) or RNAlater™ (Sigma‐Aldrich, St. Louis, MO) for downstream analysis. The wounds were closed with 2‐0 Prolene® sutures (Johnson and Johnson, New Brunswick, NJ) using an interrupted horizontal mattress suture technique with alternating directions for each closure. The four most posterior wounds were closed on day 0, post initial surgery. A DuoDerm® dressing (ConvaTec, Bridgewater, NJ) was used to form a perimeter around each wound array. All remaining open wounds were plugged with a pre‐cut GranuFoam™ dressing (1 cm long × 1 cm wide × 2 cm deep) (Kinetic Concepts Inc. [KCI] an Acelity company, San Antonio, TX). A TheraBond® 3D Antimicrobial System dressing (Argentum Medical LLC, Geneva, IL) with pre‐cut windows was placed around each wound array enabling the GranuFoam™ plugs to protrude through. No systemic antibiotics were used. A GranuFoam™ pad was placed over each wound array so that all protruding GranuFoam™ plugs interfaced directly with the GranuFoam™ pad. A Tegaderm™‐like VAC adhesive drape was placed over each wound array so that it completely covered the wound array and DuoDerm® dressing. A 2.5 cm hole was cut in the centre of each VAC drape, and a VAC port was attached. A VAC VIA™ pump (KCI/Acelity) was attached to the right side of the animal, and 125 mmHg was applied at a constant rate. The left side of each animal served as the control. The animal was placed in a custom‐made protective jacket (Lomir Biomedical Inc., Notre‐Dame‐de‐l'île‐Perrot, Quebec, Canada) to protect the wounds and hold the vacuum pump. The VAC VIA™ pump was changed out every 8 hr on each animal. The surgery was repeated again at 3, 6 and 9 days post initial surgery. The same size GranuFoam™ piece was inserted in the wounds at each dressing change and was not decreased in size even if the wound was decreasing in size as evidenced by a change in wound perimeter. Four wounds were excised containing GranuFoam™ at each time point by making an elliptical cut around each wound that was approximately 1.5 cm deep and 1.5 cm wide with a scalpel. All excised tissue was bisected and preserved in 10% NBF and stored at 4°C for at least 1 week or RNAlater™ (Sigma‐Aldrich) and refrigerated at 4°C for 24 hr followed by storage at −80°C. After excision, tissues were sutured closed using the same procedures as the prior surgery. Wounds were excised from posterior to anterior over time. At each time point, open wounds were re‐plugged with fresh GranuFoam™. Animals were euthanized while under deep level general anaesthesia via exsanguination. An overview of the entire experimental procedure is depicted in Figure 1A–M.

### 2.3 Histological analysis of pig tissue explants

Samples preserved in 10% NBF were removed from 4°C storage then washed with phosphate buffered saline thrice and placed in 70% ethanol for at least 24 hr. Samples were sent to the KUMC histology core for paraffinization. Samples were then collected and sent to Charles River, where samples were serial sectioned at a thickness of 10 μm and stained with hematoxylin & eosin (H&E), Masson's Trichrome and Brown & Brenn (modified gram stain) in a repeating pattern on adjacent sections. Charles River followed in‐house protocols for all sets of stains. H&E stains cytoplasm, matrix and plasma proteins (fibrin[ogen], fibronectin, vitronectin) pink and cellular nuclei dark purple. Masson's Trichrome stains matrix and plasma proteins red, collagen blue and cellular nuclei black. Additional staining was performed in‐house utilizing Picrosirius Red (Cat# ab150681, Abcam, Cambridge, UK) staining with polarized microscopy and Alcian Blue (Cat# 8378, ScienCell, Carlsbad, CA) staining to further characterize collagen composition and glycosaminoglycans (GAGs), respectively. Picrosirius Red staining was carried out per the manufacturer's protocol. Under polarized light, collagen type I appears red and collagen type III appears green. Alcian Blue staining was carried out per manufacturer's protocol, with sulphated GAGs staining blue, nuclei red and cytoplasm pink.

### 2.4 Tissue thickness measurements

**FIGURE 2:**
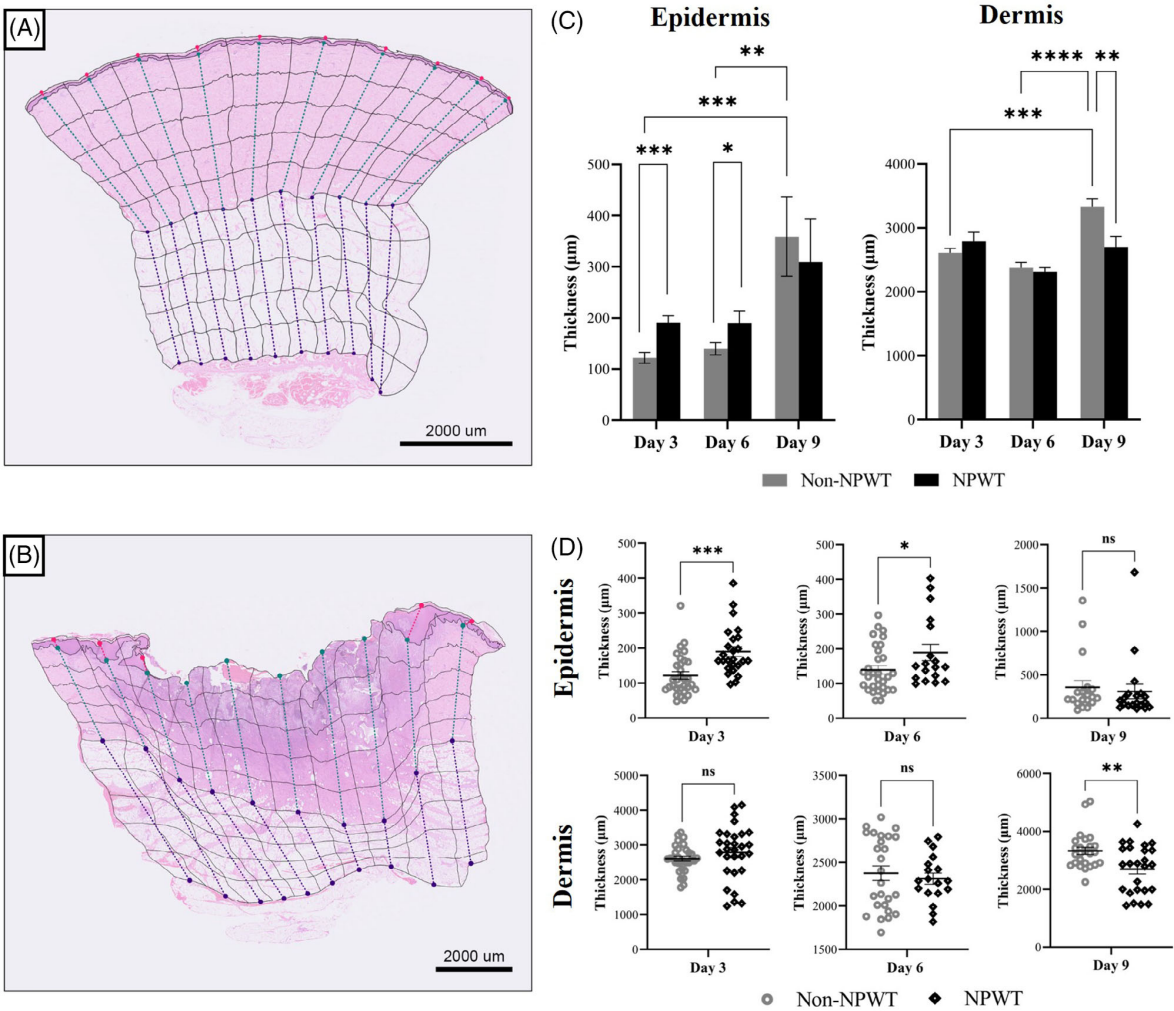
NPWT modulates thickness of skin layers. (A, B) Reconstructed H&E stained tissue sections from 200x total magnification images with coloured, dashed lines indicating where measurements took place wherein (A) is baseline tissue control biopsy and (B) is injured tissue after 9 days of recovery. The thickness of the epidermis and dermis, for non‐NPWT and NPWT, is shown in graphical form to the right. (C) Non‐NPWT was directly compared to NPWT for temporal trends over days 3, 6 and 9 for the epidermis and dermis. Grey bars indicate non‐NPWT wounds. Black bars indicate NPWT wounds. (D) Each individual day and layer were then divided into individual scatterplots to demonstrate distribution of measurements that compared non‐NPWT to NPWT. Grey circles indicate non‐NPWT wounds. Black diamonds indicate NPWT wounds. Error bars are denoted as SEM. Significance is denoted as *p < 0.05, **p < 0.01, ***p < 0.001, ****p < 0.0001, or ns for *p* > 0.05 and *n* = 4. Scale bar =  2000 μm

Unique regions of interest (ROIs) gridding was developed to account for the natural curvature and contour of wounded soft tissue (Figure 2A,B). For each column of ROIs in a given tissue layer, the midpoints of the topmost ROI and the bottom most ROI were connected, and a straight line connecting the ends of the midpoints was measured and recorded. The thickness of the tissue layers can vary considerably across the width of the sample, particularly in wounded tissue, which makes single point sampling of a given layer less than accurate. To offset this variability, 10 thickness measures per layer per tissue section were collected for all four wounds of each given treatment group, for a total of up to 40 possible measurements (Figure 2).

### 2.5 Histological quantification of immune cell wound infiltration

**FIGURE 3:**
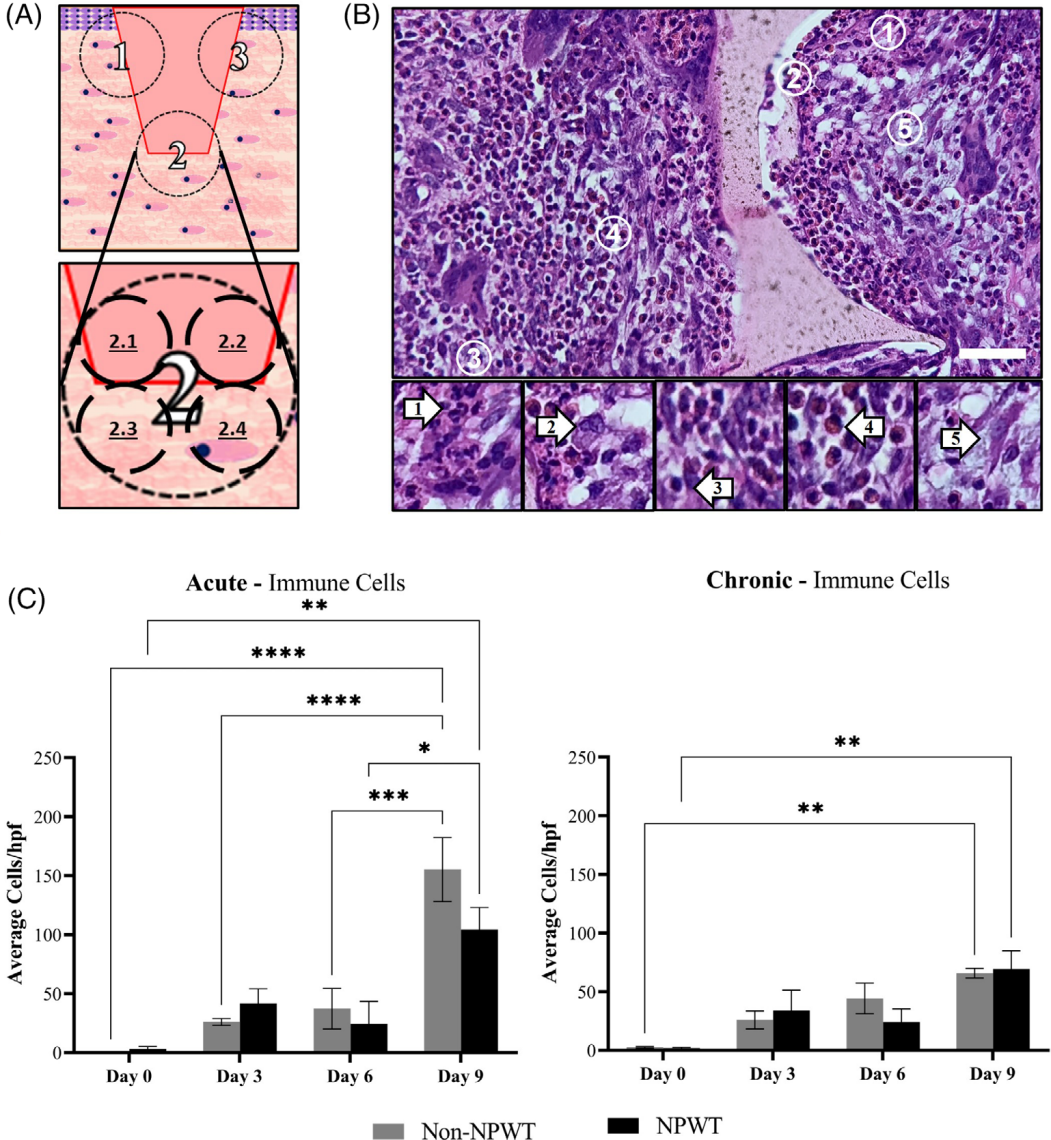
No change in relative immune cell populations with NPWT. Tissue explanted samples at day 0, 3, 6 and 9 were stained with H&E and analysed under light microscopy for immune cell population analysis. (A) When looking at each H&E stained slide, each wound sample was divided into three low magnification regions indicated by the black dashed circles labelled 1, 2 and 3. Within each of the three low magnification regions, four high‐powered magnification regions were obtained based off of immune cell density. Purple cells are the epidermal cells, pink cells are dermal cells and red quadrilateral shape is the wound. (B) A 200× H&E image displaying immune cell infiltrate containing neutrophils (1), macrophages (2), lymphocytes (3), eosinophils (4) and fibroblasts (5). Lower panel of five high magnification images of each numbered circles from low magnification image (*above*) to denote which individual cell labelled. (C) Bars graphs denoting the average acute (*left*) immune cell population per ‘hpf’ and average chronic (*right*) immune cell population per ‘hpf’. Acute and chronic immune cell populations were identified from each of the four high magnification (40× objective with 10× eye piece; hpf) regions from each of the three low magnification (4× objective with 10× eye piece) regions. Grey bars indicate non‐NPWT wounds. Black bars indicate NPWT wounds. The *y*‐axis is number of cells per ‘hpf’. Error bars are SEM. Significance is denoted as *p < 0.05, **p < 0.01, ***p < 0.001, or ****p < 0.0001 and *n* = 4. Scale bar = 50 μm

Analysis of the immune cell infiltration into the wound site was performed on tissue explants at days 0, 3, 6 and 9. Wounds were either treated with full wound dressings and subatmospheric pressure (i.e., NPWT) or wound dressings without subatmospheric pressure (i.e., non‐NPWT). Day 0 excisional wounds not exposed to therapy or dressings were used as a baseline for tissue comparison. A total of two wounds per experimental group were obtained from each pig giving a total of four samples per experimental group. Samples were sectioned and stained for H&E. The H&E slides were provided to a blinded clinical dermatopathologist, who analysed the slides for presence of acute and/or chronic immune cell populations via light microscopy using an Olympus BX46 microscope, (Olympus, Center Valley, PA). Histological analysis of the wounds demonstrated heterogeneity in their shapes, sizes and overall appearance. Therefore, in order to standardize the approach of cell number quantification, an array method was developed to calculate cell numbers from H&E slides at ‘easy to identify’ anchor points within the tissue. Analysis consisted of dividing the general wound structure into three regions (one region at each side of the wound at the dermal‐epidermal junction and one region at the basal surface of the wound; see Figure 3A) at low power objective (4× objective and 10× eyepiece). The three lower magnification regions were then further subdivided into four higher power objective (40× objective and 10× eyepiece) regions. These higher magnification regions were determined by taking the most densely populated regions within 1 mm of each of the three regions. The higher magnification regions were counted for both acute immune cells (neutrophils) and chronic immune cells (lymphocytes, macrophages and eosinophils), independently. A total of four high powered fields (hpf) of view were attempted to be counted for each of the three regions to obtain an average for up to 12 total counts per wound (3 regions [low mag] × 4 counts [high mag] = 12 total). Wound groups were performed in duplicates for each pig (12 counts × 2 replicates = 24 total) and a total of two pigs were used, totalling for up to 48 total counts for each experimental wound group. The 12 total counts per wound group of each pig were added together to form an aggregate average of the wound site inflammation. Each of the averages for the NPWT treatment groups were compared to the non‐NPWT counterparts and indicated as total number of immune cells per ‘hpf’. This was done for both acute and chronic cells. Due to the delicacy of tissue samples during wound healing and sample processing with the foam in situ, some samples were torn or lost a portion of tissue during processing and four measurement per ‘hpf’ was not always feasible.

### 2.6 Gene expression and analysis

When ready for processing, tissue explants were bisected with a vertical cut via a scalpel to split the excised wound tissue into equal halves that contained the full epidermis, dermis and subcutaneous tissue layers. The GranuFoam™ was removed from each tissue sample to allow for maximum RNA isolation. Samples were weighed and tissues were trimmed outside the wound edges with a scalpel until each sample weighed 30 mg for RNA isolation. RNA was isolated and purified from tissue samples using a RNeasy Mini Kit (Qiagen) according to manufacturer's instructions. RNA integrity was assessed using an Agilent Bioanalyzer (Agilent Technologies, Santa Clara, CA). Samples that displayed an RNA integrity number of 7 or greater were used for downstream processing. Samples were reversed transcribed using High Capacity cDNA Reverse Transcription Kits (ThermoFisher Scientific, Waltham, MA) and a qTower^3^ real‐time thermocycler (Analytik Jena, Jena, Germany) according to manufacturer's instructions. Samples were analysed for purity using a QuickDrop micro‐volume spectrophotometer (Molecular Devices, San Jose, CA). Samples that displayed an absorbance ratio (*A*_260_/*A*_280_) of 1.8 were designated pure and used for analysis. Gene expression was assessed using real‐time quantitative polymerase chain reaction (RT‐qPCR) using a qTower^3^ real‐time thermocycler. A Qiagen *RT* ^2^ Profiler™ PCR Array for Pig Wound Healing (PASS‐121ZC‐24) was used to assess for genomic expression of 84 wound healing genes. Cycle threshold (*Ct*) values were recorded and analysed via the Delta–Delta–*Ct* method. Glyceraldehyde 3‐phosphate dehydrogenase (GAPDH), beta‐actin (ACTB), hypoxanthine phosphoribosyltransferase‐1 (HPRT1) and ribosomal protein L13a (RPL13A) were the endogenous control genes utilized by the array. Excision of day 0 biopsies used to inflict initial wounds were used as the baseline tissue control for which each NPWT and non‐NPWT sample *Ct* values were compared against to calculate the relative change in gene expression.

### 2.7 Statistical analysis

All data are reported as means with SEM. A power analysis indicated that a minimum of 10 pigs were needed to perform appropriate statistical tests. However, due to the nature of this study being a ‘pilot study’ only two pigs were utilized. To allow for statistical tests to be performed, wounds were performed in duplicate for each pig, providing a total of four (*n* = 4) wounds for each treatment group. Histological analysis of immune cell populations and all genomic analyses were assessed using a two‐way ANOVA approach. Histological analysis of skin layer thickness measurements utilized a two‐way ANOVA for assessing the differences temporally. For scatter plots of skin layer thickness for each individual time point, an unpaired student's *t*‐test was used.

## 3 RESULTS

### 3.1 NPWT exposure modulates thickness of skin layers

The thickness of the epidermal and dermal layers of skin can vary depending on a variety of stimuli, including hyperproliferation, inflammatory infiltration, edema and fibrosis. Modulation of layer thickness for the epidermis and dermis was assessed by generating a unique ROI grid of the H&E‐stained tissue sections (Figure 2A,B). Analysis demonstrated that NPWT resulted in a significant increase in epidermal thickness at days 3 and 6, when compared to non‐NPWT (Figure 2C,D). Conversely, exposure of NPWT to wounds did not result thickening of the dermal layer, whereas non‐NPWT wounds had increased dermal thickening. This can be seen at day 9 in the non‐NPWT wounds where there was a significant increase relative to days 3 and 6 in the non‐NPWT wounds, in addition to NPWT wounds on day 9 (Figure 2C,D).

### 3.2 No change in relative immune cell populations with NPWT

Analysis of the immune cell infiltration into the wound site was performed on both non‐NPWT and NPWT wounds. The average of each of the wounds ‘hpfs’ was obtained (Figure 3C). The data demonstrated a significant increase of both acute and chronic immune cell populations by day 9 for both the non‐NPWT‐ and NPWT‐treated wounds. Overall, no significant differences were noted between non‐NPWT‐ and NPWT‐treated wounds for either cell population at any of the time points in this study.

### 3.3 NPWT enhances plasma protein adsorption and matrix deposition within GranuFoam™

**FIGURE 4:**
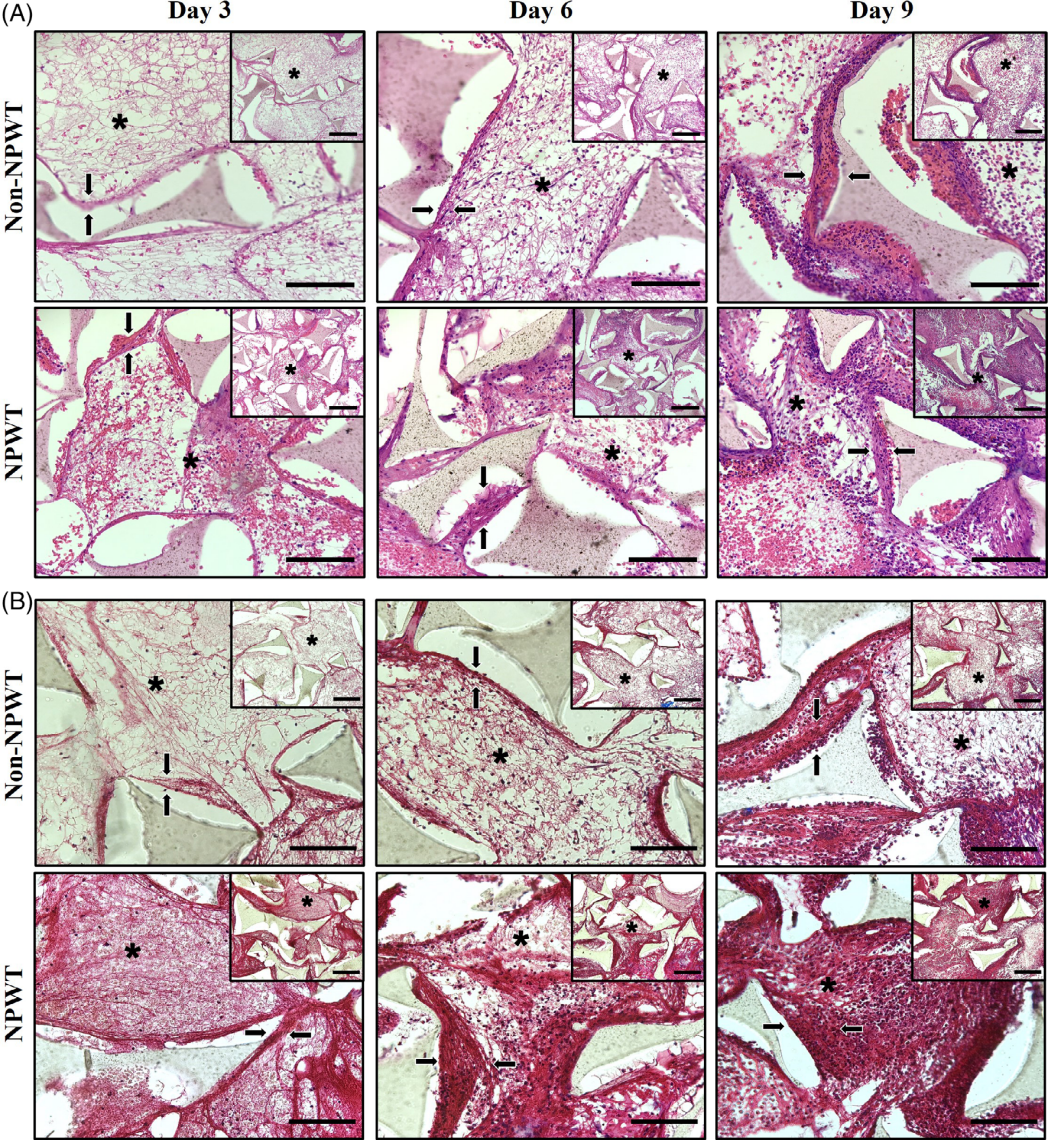
NPWT enhances plasma protein adsorption and tissue enmeshing within GranuFoam™. Tissue samples explanted from pigs at day 3, 6 and 9 were histologically stained and analysed under light microscopy. Regions within most interior portion of GranuFoam™ were assessed to decrease impact of ‘enmeshing’ from wound edges. (A) H&E and (B) Masson's Trichrome images at 200× magnification comparing non‐NPWT (*top row*) and NPWT (*bottom row*) over the time points of day 3 (*first column*), day 6 (*second column*) and day 9 (*third column*). Inset is image at 100× magnification. Protein adsorption onto GranuFoam™ denoted by arrows. Open network of pores within GranuFoam™ denoted by black ‘*’ and corresponds to ‘*’ in inset image. Residual GranuFoam™ can be seen as a ‘multi‐pointed particulate’ debris residing within the wound bed. H&E staining evaluates ECM proteins (i.e., collagen) and plasma proteins (i.e., fibrinogen) and is identified as light pink. Dark purple staining represents cellular nuclei. Masson's Trichrome staining evaluates for collagen by staining blue. Plasma proteins and non‐collagen matrix proteins stain red. Nuclei are stained dark purple/black. It is important to note that each image represents GranuFoam™ in the wound for the same amount of time (i.e., 3 days). Scale bar = 100 μm for 200× and 200 μm for 100× (inset)

Histological sections stained for H&E and Masson's Trichrome were used to analyse the interaction of the healing wound tissue with GranuFoam™ in situ for each 3‐day timepoint. Regions towards the interior portion of GranuFoam™ were assessed for protein adsorption upon GranuFoam™ to decrease interference of ‘enmeshing’ from wound edges. The H&E sections demonstrated an increasing trend in plasma protein deposits (pink) within the porous network of the GranuFoam™ from day 3 to day 9 in both the non‐NPWT and NPWT wounds (Figure 4A). Similarly, a thicker protein deposition can be seen adsorbing to the surface of GranuFoam™ particles over time for each group (*Note: tissue processing can result in protein layer detaching from GranuFoam™ leaving a void space*). However, NPWT resulted in more abundant protein deposition between and onto GranuFoam™ particles at each time point (Figure 4A). Notably, the H&E sections revealed enhanced immune cell localization (dark purple) within the dense protein deposits around GranuFoam™ particles for both non‐NPWT and NPWT wounds (Figure 4A).

The Masson's Trichrome samples showed a similar trend as the H&E staining. There is an increasing deposition of matrix and plasma proteins (red) within the porous network of the GranuFoam™ as the wounds evolved over time in both non‐NPWT and NPWT (Figure 4B). Similarly, there was enhanced deposition of matrix and plasma proteins adsorption onto GranuFoam™ particles. Wounds exposed to NPWT had more abundant protein deposition between and onto GranuFoam™ particles at every time point, relative to non‐NPWT (Figure 4B). As shown with the H&E sections, increased cellular localization is seen depositing onto GranuFoam™ particles (Figure 4B). Additionally, enhanced tissue ingrowth (i.e., enmeshing) from the wound bed/edges into the GranuFoam™ is seen (Figure S1). Both non‐NPWT and NPWT wounds demonstrated an increasing trend in collagen type I deposition (blue) temporally, as well as collagen type III (green) (Figure S2). Overall, NPWT appeared to increase enmeshing to a greater extent in both density and penetration depth.

Additional staining was performed to further characterize composition of the deposited tissue by staining with Alcian Blue and Picrosirius Red, for characterization of sulphated GAGs and collagens, respectively. Day 9 wounds further demonstrated encapsulation of GranuFoam™ particles with GAGs (blue) and collagen type I (red/orange) and collagen type III (green) (Figure S2). Interestingly, day 9 wounds exhibited a leading edge of GAGs at the base of the wound bed and around GranuFoam™ particles in both groups. However, NPWT appeared to result in more abundant GAG deposition overall.

### 3.4 NPWT limits dissemination of bacteria to adjacent tissue

**FIGURE 5:**
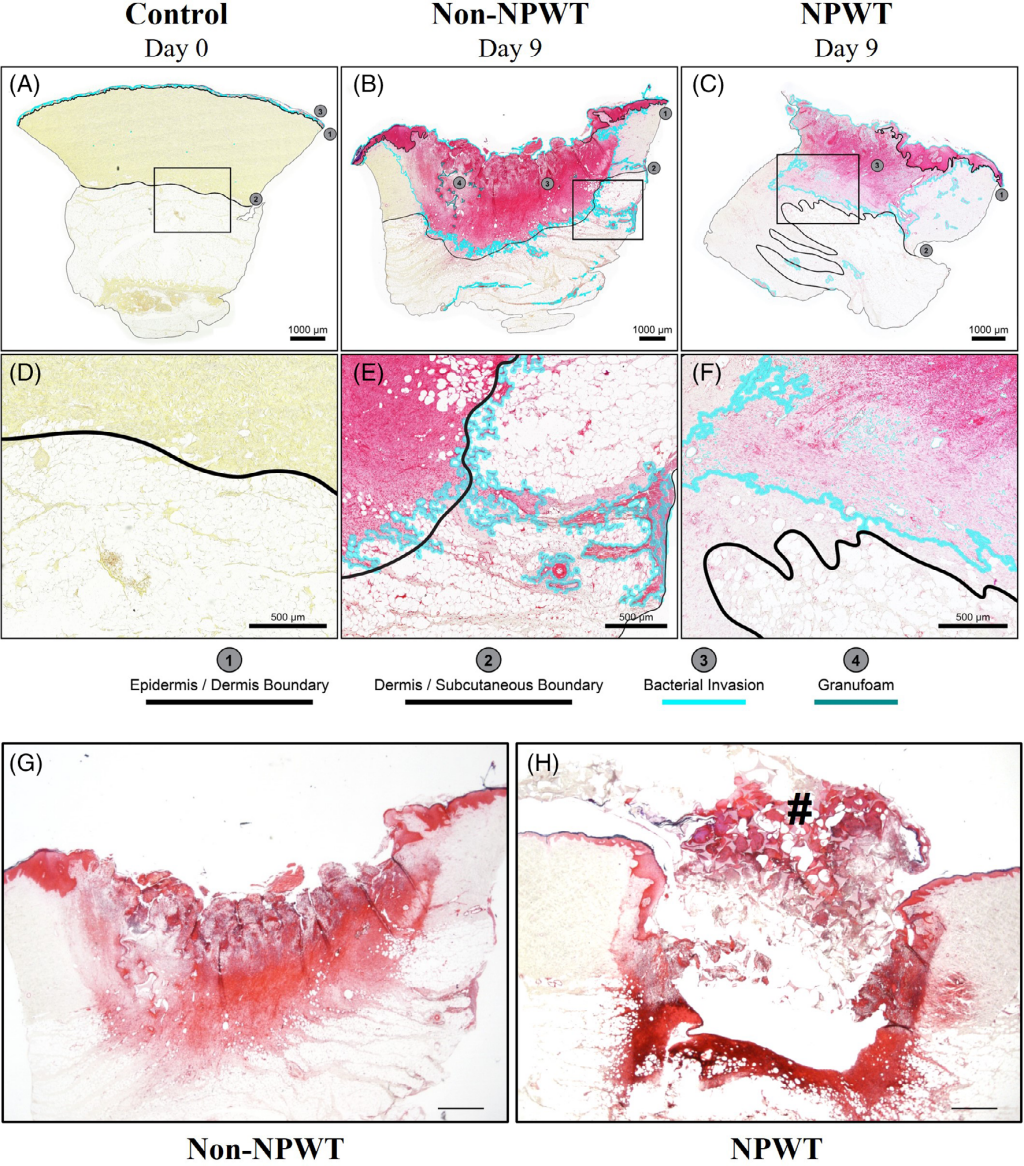
NPWT limits dissemination of bacteria to adjacent tissue. Tissue samples explanted from pigs at day 0, 3, 6 and 9 were stained with the Brown & Brenn method and analysed with high resolution imaging software and montaged together. Gram positive bacteria stain purple. Gram negative bacteria stain pink/red. Background tissue stains light yellow. (A–F) Demonstration of bacterial invasion on day 9 of wound (B,E) non‐NPWT and (C,F) NPWT. (A–C) Low magnification images of entire wound. Solid black line #1 depicts the epidermal/dermal junction. Solid black line #2 depicts dermal/subcutaneous junction. Light blue line #3 is an artificial addition to the image to depict furthest extent of bacterial invasion. Dark blue line #4 is depicting GranuFoam™ (D–F) Depicts high magnification of region highlighting furthest extent of bacterial invasion. (A,D) Day 0 excisional biopsies were used as a baseline tissue control comparison and indicated gram positive and negative bacteria. (B,E) Day 9 non‐NPWT wounds. In wounds without NPWT treatment, gram negative bacteria infiltrated into the subcutaneous layer, indicated by more diffuse and darker pink/red stain. (C,F) Day 9 NPWT wounds. In wounds treated with NPWT, the gram negative bacteria was found to be most dense around the remaining GranuFoam™, with limited dissemination to adjacent tissue, relative to non‐NPWT wounds. Day 9 (G) non‐NPWT and (H) NPWT wounds are shown to further depict bacterial localization. In the NPWT, a bacteria‐laden GranuFoam™ dressing can be seen coming out of the wound bed (denoted by black ‘**#**’). Scale bar = 1000 μm

Tissue sections were further analysed for bacterial localization via a modified Gram stain (Brown & Brenn) to assess bacterial presence. Gram negative bacteria are stained pink/red, and gram positive bacteria are stained deep purple. There is limited gram positive bacteria at the superficial surface of the skin making up the skin flora but with minimal gram positive bacteria present as a whole (Figure 5A–H). Conversely, there is a more abundant source of gram negative bacteria found within the wound and adjacent tissue locations within the dermis and subcutaneous regions. Overall, gram negative bacterial burden is more diffusely spread in the non‐NPWT wounds, infiltrating to a greater extent away from the wound bed/edge into the dermis and subcutaneous regions (Figure 5B,E,G). Wounds exposed to NPWT resulted in a more abundant localization of bacteria within the GranuFoam™, with less dissemination into adjacent tissue regions (Figure 5C,F,H). Notably, an image of a NPWT treated wound at day 9 demonstrating a dense bacteria‐laden dressing detaching from the wound bed can be seen in Figure 5H.

### 3.5 Genomic wound healing profile

**FIGURE 6:**
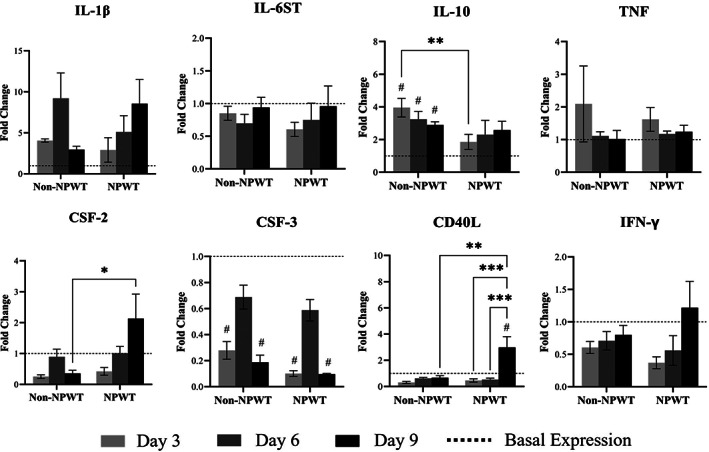
Inflammatory genomic profile of wound healing. Elliptically explanted wound tissue was assessed for expression of key genes via a wound healing array. Significant genes involved with the inflammatory process of wound healing were grouped together and analysed at days 3, 6 and 9 post‐injury, relative to baseline tissue controls. Values are reported as fold change against their respective gene expression to baseline tissue biopsies and normalized to a group of endogenous control genes, that included GAPDH, ACTB, HPRT1 and RPL13A. Each graph compares intragroup temporal differences and intraday difference between non‐NPWT and NPWT. Non‐NPWT (*left set*) and NPWT (*right set*) average fold changes are depicting temporally with day 3 (*light grey*), day 6 (*dark grey*) and day 9 (*black*). A dashed line at a value of ‘1’ is used to depict average baseline expression. Error bars are SEM and include *n* = 4. Significance on non‐NPWT and NPWT wounds relative to the baseline tissue is denoted with a ‘**#**’ above bar and indicates a *p* < 0.05. Intragroup and intraday significance is denoted as *p < 0.05, **p < 0.01 or ***p < 0.001 and *n* = 4

Tissue isolated from each wound was assessed for changes in expression of wound healing genes via a wound healing array. Expressional changes were first compared to baseline tissue controls (denoted as ‘#’ if significant) and then compared temporally for intragroup differences (i.e., NPWT day 3 vs. NPWT day 9), in addition to comparing intraday differences between groups for each time point (i.e., NPWT day 9 vs. non‐NPWT day 9). Expressional changes in key genes involved in inflammation are shown in Figure 6, which demonstrated similar responses in non‐NPWT and NPWT wounds when comparing to basal tissue, including both intraday comparisons and temporal trends within groups. Both non‐NPWT and NPWT exhibited a significant decrease from basal tissue for CSF3 (also known as G‐CSF) on days 3 and 9 but not day 6. Significant differences between non‐NPWT and NPWT are seen in IL10, CSF2 (also known as GM‐CSF) and CD40L. IL10 is significantly increased from basal tissue expression in non‐NPWT, whereas NPWT does not result in a significant increase. CSF2 demonstrated a significant increase at day 9 for NPWT compared to non‐NPWT on day 9. Similarly, expression of CD40L is significantly increased on day 9 for NPWT, relative to all intragroup and intraday comparisons.

**FIGURE 7:**
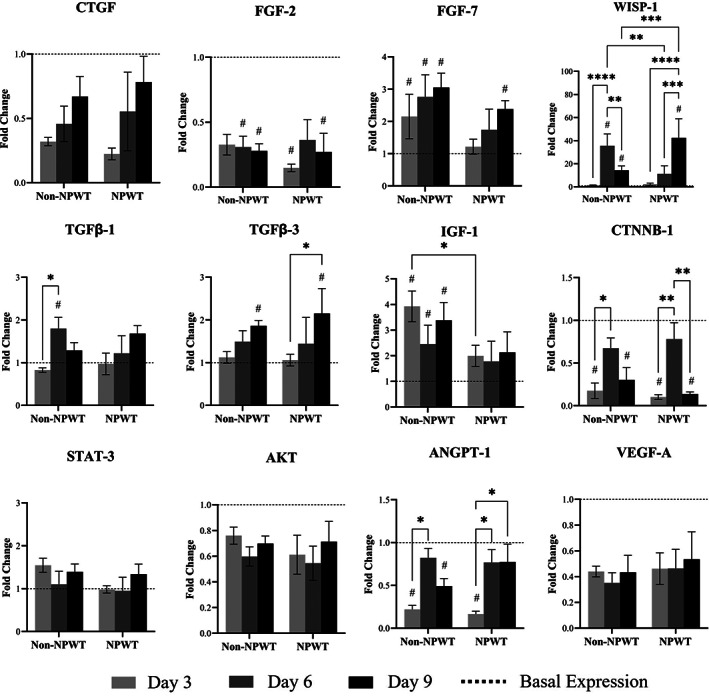
Mitogenic genomic profile of wound healing. Elliptically explanted wound tissue was assessed for expression of key genes via a wound healing array. Significant genes involved with the mitogenic process of wound healing were grouped together and analysed at days 3, 6 and 9 post‐injury, relative to baseline tissue controls. Values are reported as fold change against their respective gene expression to baseline tissue biopsies and normalized to a group of endogenous control genes that included GAPDH, ACTB, HPRT1 and RPL13A. Each graph compares intragroup temporal differences and intraday difference between non‐NPWT and NPWT. Non‐NPWT (*left set*) and NPWT (*right set*) average fold changes are depicting temporally with day 3 (*light grey*), day 6 (*dark grey*) and day 9 (*black*). A dashed line at a value of ‘1’ is used to depict average baseline expression. Error bars are SEM and include *n* = 4. Significance on non‐NPWT and NPWT wounds relative to the baseline tissue is denoted with a ‘**#**’ above bar and indicates a *p* < 0.05. Intragroup and intraday significance is denoted as *p < 0.05, **p < 0.01, ***p < 0.001, or ****p < 0.0001 and *n* = 4

Another important stimulus for wound healing is mitogenic signalling, which includes a variety of growth factors and other proliferative markers. The mitogenic expressional profile is shown in Figure 7. Again, globally there is similar response patterns in non‐NPWT and NPWT compared to basal tissue. When comparing both non‐NPWT and NPWT wounds to basal tissue, there is a significant decrease in FGF2 and ANGPT1, whereas there is a significant increase in FGF7, WISP1, TGFβ3 and CTNNB1. Notably, there is an increase in TGFβ1 and IGF1 only for non‐NPWT, compared to basal tissue, but not NPWT wounds. Both non‐NPWT and NPWT had a significant intragroup increase of ANGPT1 on day 6, though only NPWT sustained that increase on day 9. Similarly, there is an increase of CTNNB1 on day 6 compared to day 3 for both non‐NPWT and NPWT. The only genes to show significant intraday differences between non‐NPWT and NPWT were WISP1 and IGF1. WISP1 showed an increase on day 6 for non‐NPWT, whereas on day 9 WISP1 was higher in NPWT. IGF1 had a significantly higher expression on day 3 in non‐NPWT when compared to NPWT.

**FIGURE 8:**
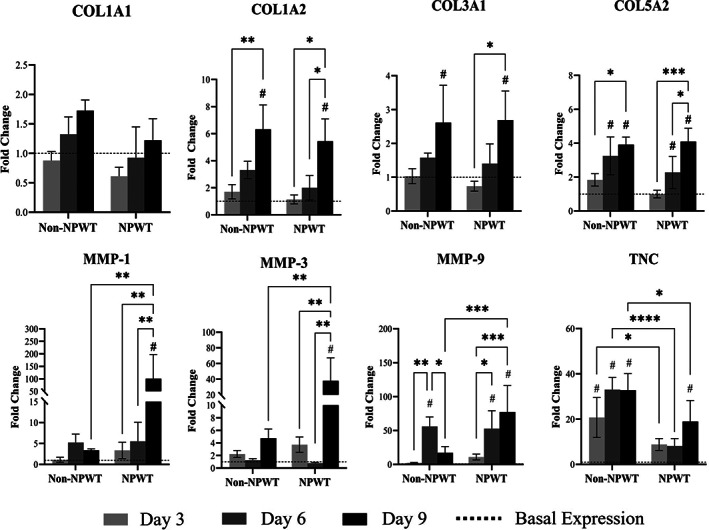
ECM remodelling genomic profile of wound healing. Elliptically explanted wound tissue was assessed for expression of key genes via a wound healing array. Significant genes involved with the ECM remodelling process of wound healing were grouped together and analysed at days 3, 6 and 9 post‐injury, relative to baseline tissue controls. Values are reported as fold change against their respective gene expression to baseline tissue biopsies and normalized to a group of endogenous control genes that included GAPDH, ACTB, HPRT1 and RPL13A. Each graph compares intragroup temporal differences and intraday difference between non‐NPWT and NPWT. Non‐NPWT (*left set*) and NPWT (*right set*) average fold changes are depicting temporally with day 3 (*light grey*), day 6 (*dark grey*) and day 9 (*black*). A dashed line at a value of ‘1’ is used to depict average baseline expression. Error bars are SEM and include *n* = 4. Significance on non‐NPWT and NPWT wounds relative to the baseline tissue is denoted with a ‘**#**’ above bar and indicates a *p* < 0.05. Intragroup and intraday significance is denoted as *p < 0.05, **p < 0.01, ***p < 0.001 or ****p < 0.0001 and *n* = 4

Lastly, expressional changes in extracellular matrix (ECM) remodelling genes were assessed and are shown in Figure 8. Expressional patterns for different collagens were similar between both non‐NPWT and NPWT, compared to basal tissue. There was a significant increase temporally from day 3 to day 9 in both non‐NPWT and NPWT for COL1A2 and COL5A2, in addition to a significant increase in COL3A1 for NPWT only. Notably, there was a significant increase in expression of multiple proteases on day 9 for NPWT relative to both basal tissue and non‐NPWT wounds, including MMP‐1, MMP‐3 and MMP‐9. Whereas a decrease in TNC is seen in NPWT compared to non‐NPWT for every time point. Interestingly, expression of COL1A2, COL3A1 and MMP‐3 are all significantly increased when comparing the day 0 wounds to the day 0 baseline tissue biopsies (Figure S3), elucidating to the early signalling (within 60 min of wounding) responses potentially important for later downstream signalling responses seen.

### 3.6 GranuFoam™ induces FBR

**FIGURE 9:**
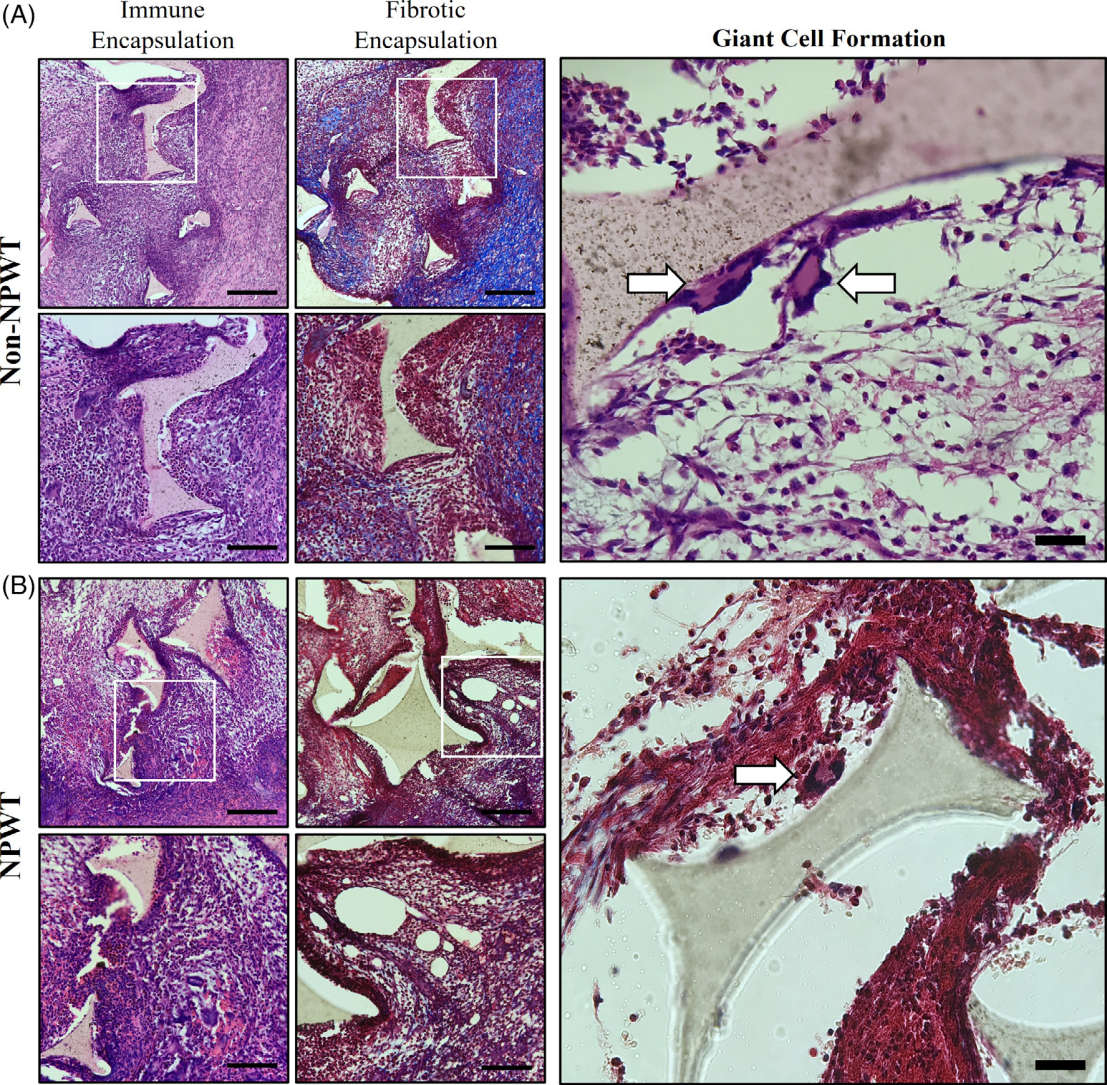
GranuFoam™ induces FBR. Tissue samples explanted from pigs at day 9 were histologically stained and analysed under light microscopy. Regions within/around wound bed were assessed. Characterizing (A) non‐NPWT and (B) NPWT at day 9 with H&E (*left column*) and Masson's Trichrome (*middle column*) images at 100× (*top rows*) and 200× (*bottom row*) magnification for presence of a FBR. A 400× magnification image of giant cells (*white arrows*) upon GranuFoam™ within the wound are also shown (*large rightmost panels*). H&E images demonstrate waves of fibrous matrix material (pink) and dense chronic immune cell deposits around GranuFoam™. Masson's Trichrome images demonstrates similarly, waves of dense fibrous material of different matrix components including collagen (blue) and other matrix‐derived components (red) encapsulating the GranuFoam™. Scale bar = 50 μm for 400×, 100 μm for 200× and 200 μm for 100×

The in situ viewpoint paired with use of GranuFoam™ dressed wounds without subatmospheric pressure exposure (non‐NPWT) provided a novel perspective to visualize the presence of a FBR to the GranuFoam™ dressing material within wounds. This is shown histologically with the H&E and Masson's Trichrome stained sections (Figure 9A,B). The GranuFoam™ particles are seen encapsulated by dense chronic immune cell populations including macrophages/histiocytes, eosinophils and lymphocytes. These cells are intermixed with fibroblasts and accompanied by deposits of fibrous matrix (H&E—pink, Trichrome—blue/purple/red) around the GranuFoam™. This response is seen in both non‐NPWT and NPWT (Figure 9A,B). Additionally, to further support the presence of a FBR, there is formation of giant cells (arrows) in non‐NPWT and NPWT wounds, both within the wound and within the wound bed, accompanied by neovascularization (Figure S10).

## 4 DISCUSSION

In this study, new insight was provided for wound healing in the context of NPWT utilizing the wound VAC system. The VAC system was a landmark development in the field of wound healing and has provided clinicians the ability to accelerate the wound closure process in a variety of applications.^26^, ^31^ The VAC has proven advantageous in difficult to treat and non‐healing wounds.^42^, ^43^ Previous studies have demonstrated that the VAC is able to augment local blood flow, reduce edema and remove exudative fluids within the wound site.^25^, ^44^, ^45^ The VAC is thought to enhance blood flow by promoting fluid egress from the wound and decreasing interstitial pressure on the microvasculature, resulting in a decrease in capillary afterload within the tissue.^45^, ^46^ The VAC has also been shown to enhance the rate of granulation tissue deposition, which is considered to play a significant role in wound healing outcomes seen with the VAC system due to matrix deposition and the highly vascular nature of granulation tissue.^25^, ^26^ One suggested mechanism for the increased rate of granulation tissue deposition is thought to be due to mechanotransduction signalling, which results when an external force leads to structural interactions between the ECM and cellular cytoskeleton.^47^ Mechanotransductive signalling then results in enhanced mitotic activity and global protein production.^46^, ^47^, ^48^ Additionally, enhanced neovascularization within granulation tissue is thought to result in greater perfusion to the tissue, with subsequent increases in neutrophilic response and antibacterial activity with the VAC^25^

Overall, the VAC system is well known for its ability to modulate wound healing and its clinical outcomes are well‐documented.^42^, ^49^, ^50^, ^51^ However, studies are yet to clearly demonstrate mechanistically how the VAC system modulates wound healing at a genomic and proteomic level that correlates to what is seen clinically. Specifically, how exposure to subatmospheric pressure augments tissue deposition, inflammation and the overall wound healing process. To our knowledge, no study to date has looked at an in situ perspective with VAC therapy or utilized a GranuFoam™ dressed wound without subatmospheric pressure to assess the wound healing response in the presence of the GranuFoam™ dressing alone. Moreover, the effect of wound reinjury via repetitive removal and reapplication of GranuFoam™ to wounds should be clarified. Thus, elucidation to the dynamic mechanism(s) of wound environment augmentation, enhanced granulation tissue deposition and bacterial mitigation with the VAC, remains in question.

In this study, augmentation of the immune response was assessed histologically by quantifying acute (neutrophils) and chronic (lymphocytes, macrophages and eosinophils) immune cell populations within the GranuFoam™ and surrounding tissue in situ. A temporal increase was observed in both acute and chronic cell populations, though no differences were noted overall between non‐NPWT and NPWT. This data is contrary to previously suggested antimicrobial effects of NPWT. Indicating that the increases in neutrophil response in previous studies is potentially not a direct result of NPWT. Instead, the equivalent immune cell response seen is more likely a result of a stimulus that is consistent between the non‐NPWT and NPWT groups.

One such consistent event occurring in both non‐NPWT and NPWT wounds is the repetitive insertion of GranuFoam™ and dressing changes that occurred every 3 days. The repetitive removal of the GranuFoam™ likely induces a mechanical insult to the healing tissue due to enmeshing. In this study, we demonstrate matrix deposition within the GranuFoam™ dressing, including collagens type I and type III for both non‐NPWT and NPWT wounds (Figures S1 and S2). Consequently, upon removing the GranuFoam™, not only is the dressing removed but tissue enmeshing leads to subsequent removal of fresh tissue within the wound bed, reinjuring the wound. Notably, NPWT did appear to increase the relative abundance of matrix deposition and plasma protein adsorption around and upon the GranuFoam™, providing a substrate for bacterial colonization. It was due to this phenomenon that led to our hypothesis that bacterial mitigation when utilizing the VAC may be occurring via an alternative mechanism than originally thought.

The level of bacterial burden and the composition of bacteria are both important aspects when discussing a wound healing environment. Previous studies suggest that the wound VAC decreases overall bacterial burden via enhanced perfusion to the tissue and a subsequent increase in neutrophilic response.^25^ By performing in situ analysis of the GranuFoam™ dressed wounds, localization of bacterial colonies within the open‐cell meshwork of the GranuFoam™ and the surrounding tissue were visualized. This perspective allowed for the entire wound to be visualized and extent of bacterial dissemination to be directly compared between treatment groups; whereas previous studies have assessed bacterial burden via ex vivo analysis of wound biopsies, which does not allow for direct visualization of bacterial dissemination to adjacent tissue, nor does it take into account the role of the dressing material on bacterial burden. The enhanced protein deposition seen in NPWT was associated with higher abundance of bacterial localization within the GranuFoam™ dressing instead of the surrounding wound tissue. Therefore, the visualization of the bacterial‐laden GranuFoam™ might explain, in part, the apparent antibacterial properties of the VAC system, but only upon removal of the enmeshed dressing. Without removal, the bacterial‐laden GranuFoam™ may not suppress the bacterial burden to a clinically significant level and could lead to a localized infection as well as a systemic response and septicemia in a patient. This is clinically significant because bacteria can induce chronic inflammation within a wound and locally increase ROS and proinflammatory cytokines. Therefore, the mechanism of bacterial mitigation by the VAC system is of fundamental importance.

There is an increase over time in neutrophils into the wound area with NPWT. However, this increase is seen in non‐NPWT wounds as well, which is contrary to what previous studies have postulated as a possible mechanism for bacterial mitigation.^25^, ^26^, ^34^ One potential explanation for the steady increase in immune cells over time is potentially, in part, due to the repetitive irritation from dressing changes. Another possible explanation is that the GranuFoam™ dressing itself is inducing a FBR.^24^ The presence of giant cells with fibrotic and immune cell encapsulation of GranuFoam™ particles provides new insight that a FBR to the GranuFoam™ dressing may in fact have a role in outcomes seen with VAC therapy.

The in situ histology perspective in this study was able to reveal the increasing trend of protein and immune cell presence in and around the GranuFoam™ dressing in both non‐NPWT and NPWT wounds. Notably, each processed wound time point is equivalent and is the result of 3 days within the wound, and theoretically should have similar deposits of protein and immune cell populations. However, it appears there is an enhanced response at each sequential time point, with the GranuFoam™ dressing and dressing changes priming the wound bed to a new ‘baseline’ after each dressing change. Reinjury and reinsertion of a new GranuFoam™ dressing that has not yet been coated/encapsulated by proteins and cells results in a new stimulus and a more robust response from a new ‘baseline’ from the primed tissue. Our results show that although the overall immune cell response is similar in non‐NPWT and NPWT, there may be concentration of cells, protein and enmeshing within the GranuFoam ™ dressing with NPWT.

Futhermore, one of the main reasons the VAC system is used clinically is because it is thought to expedite the evolution of the wound healing process by enhancement of granulation tissue and ECM deposition, promotion of wound contraction and subsequent decreases in the size of the wound site.^25^, ^49^ This may suggest that NPWT is able to stimulate ECM production and remodelling at a greater capacity than wounds not treated with NPWT. Yet, the genomic data in this pilot study suggest that wounds treated with NPWT tend to have equivalent expressional levels of different collagens relative to non‐NPWT wounds, including COL1A1, COL1A2, COL3A1, COL4A3 and COL5A2. Additionally, there was no significant difference in ACTA2 expression after NPWT (Figure S4), compared to non‐NPWT, indicating that wound contraction via transition of fibroblasts to myofibroblasts was insignificant. However, there was a significant increase in MMP expression after NPWT, relative to non‐NPWT. On day 9, increases in MMP1, MMP3 and MMP9 can be seen. The mechanism behind this is not fully understood but MMPs such as MMP9 are intimately involved in inflammation, remodelling and epithelialization activity. Additionally, MMP9 is involved in the FBR in an attempt to expel foreign objects. FBGC formation tends to occur in the range of 7–14 days post‐exposure; therefore, the genomic changes occurring in day 9 samples could provide insight into this transition.^24^ Additionally, sulphated GAGs are known modulators of wound healing via regulation of protease activity, including MMPs, and are seen increased with NPWT at day 9 (Figure S2).^52^, ^53^

Additionally, no differences were seen in expression of STAT3 or AKT, which are pleiotropic proteins involved in a number of proliferative and bioactive pathways in wound healing, though significant changes in WISP1 expression in both non‐NPWT and NPWT were seen. WISP1 is a secreted product of the Wnt/β‐Catenin family of proteins and has a role in proliferation and tissue regeneration but can also be involved in fibrotic healing.^54^ The decrease in CTNNB1 (β‐catenin) suggests that WISP1 may be induced independent of β‐Catenin. Similarly, there was a significant decrease in FGF2 and ANGPT1 expression and an apparent decrease in VEGFA, which are key angiogenic factors. Similar results were seen in the KCI study with the VAC by Derrick et al.^55^ Conversely, our histological data demonstrates evidence of intense granulation tissue formation and neovascularization in both non‐NPWT and NPWT (Figure S10). Therefore, the neovascularization pathway of NPWT warrants further investigation with variable time points and stratified analysis of locations within the wound.

FGF7 (also known as keratinocyte growth factor) was significantly increased in non‐NWPT wounds for all time points, relative to basal tissue. This was only seen in day 9 NPWT wounds. This suggests a potential role of VAC therapy in modulating keratinocyte activity. A similar trend in IGF1 expression is seen, with non‐NPWT increased compared to basal tissue, but not NPWT. IGF1 is an important growth factor involved in multiple wound healing pathways, including proliferation and migration of fibroblast and keratinocytes.^56^ Notably, TNC has a significant drop in expression in NPWT, relative to non‐NPWT. TNC is involved in cellular migration and differentiation, including for macrophages and keratinocytes.^57^ Combining the decreased expression of FGF7, IGF1 and TNC, we see that NPWT may potentially be negatively regulating keratinocyte activity.

Interestingly, for the level of inflammation seen histologically, the relative expression of pro‐inflammatory cytokines did not appear to be significantly altered, including expression of IL1B, IL6ST, TNF and IFNG. Some genes that exhibited significant changes were IL10, CSF2, CSF3 and CD40L. IL10 is an important anti‐inflammatory cytokine and plays a pivotal role in alternative macrophage activation and polarization from an M1 to M2 phenotype. M2 macrophages are involved in tissue remodelling, granulation tissue formation, fibrosis and giant cell formation.^58^ Additionally, the day 9 surge in CD40L could be due to involvement of a number of different pathways. One of which is that CD40L is involved in angiogenic signalling via stimulating MMP9 release of endothelial progenitor cells.^59^ The CD40L/MMP9/EPC axis could explain the lack of prototypical angiogenic signalling discussed earlier. Therefore, further investigation into the role of this signalling axis with NPWT is warranted.

Since its inception in 1997, the VAC has been shown time and time again to augment the wound healing response by enhancing ECM and granulation tissue deposition, promoting neovascularization and increasing the neutrophilic response to combat bacteria. These responses are claimed to be a result of exposure to subatmospheric pressure (i.e., NPWT), as previously discussed. VAC therapy in this study demonstrated similar outcomes. However, the lack of increase in expression of collagens, angiogenic markers and immune cells with exposure to NPWT relative to non‐NPWT suggests that these prior mechanistic hypotheses about NPWT may not be the entire story. Our impression is that application of the GranuFoam™ dressing to wounds induces a FBR, ultimately resulting in a chronic inflammatory stimulus with fibrotic and immune cell encapsulation of the GranuFoam™ dressing. The FBR results in enhanced granulation tissue formation, neovascularization and immune cell infiltration of tissue. Whether exposure to NPWT augments the FBR relative to non‐NPWT is yet to be determined and warrants further investigations. However, NPWT did appear to increase matrix deposition, protein adsorption and subsequent bacterial and immune cell localization within the GranuFoam™, preventing a more dispersed infection and resulting in a more ‘tightened’ wound morphology. Upon additional histological analysis, both non‐NPWT and NPWT wounds revealed a mixed chronic inflammatory infiltrate with abundant eosinophils localized in tissue adjacent to GranuFoam™ (Figure S10). This is the first demonstration of the potential role of eosinophils in modulating the inflammatory response with VAC therapy. The presence of a foreign body potentially results in Th2 activation of eosinophils which are known to be involved in M2 polarization of macrophages.^60^ Thus, GranuFoam™ may prompt alternative activation of macrophages and subsequent giant cell formation via an eosinophil‐dependent pathway.

The insights provided by this study offer a new understanding into the fundamental workings of how the VAC system exerts its effects on wound healing. However, more studies are needed to further refine and validate these novel perspectives since this study was only a ‘proof of concept’ that utilized two animals. Larger studies with more animals and further genomic and proteomic analyses will help provide insight into the results seen in this study. Interestingly, a recently published study investigating NPWT in a porcine wound model as well briefly mentions the observation of wound filler‐associated foreign body reaction to residual dressing material left within the wounds,^61^ further supporting the FBR findings in this study.

The main limitation in this study, as mentioned above, is the limited number of subjects due to the purpose of the study being a ‘proof of concept’. To improve the rigour of the data acquired, multiple experimental techniques were used to provide a variety of perspectives including the in situ analysis of GranuFoam™, which has not been previously published to our knowledge. Moreover, the majority of prior studies on VAC‐associated wound healing models have used standard gauze dressings as a control. To our knowledge, none have used the GranuFoam™ dressing without subatmospheric pressure as a control comparison. By using this treatment control, the effect of subatmospheric exposure on wounds was isolated. An additional limitation is that the immune cell analysis for this study was performed via histological evaluation with H&E. Although this methodology is commonly used and accepted for histological immune cell analysis, further characterization of specific immune cell populations and their localization with the wound bed via immunolabelling could provide beneficial information and should be investigated in future studies. Future studies investigating other genomic markers, and the location within wounds of specific cells expressing these markers, would also help generate a more well‐rounded scientific understanding. Similarly, performing studies that assess early (0–12 hr) and late (>9 days) signalling markers could provide valuable insight into the physiological mechanisms of VAC therapy and the FBR.

In summary, there remains much to be learned about NPWT and the potential role the dressing material plays in wound outcomes. Further insight into the interaction between cells, ECM, bacteria and the wound dressing could provide understanding into the complex reparative processes of wound healing. The mechanical properties of NPWT have been postulated to improve overall wound healing. Though the role of irritation due to interval reapplication of the dressing is not adequately understood nor is the role of the tissue ‘priming’ that was demonstrated in this study. Nonetheless, dressing removal is clinically necessary to ensure proper healing and avoidance of dressing integration into a healed wound. This study demonstrates that bacteria are residing within the enmeshed GranuFoam™ dressing and that the encroachment of tissue into the dressing likely provides an advantageous environment for bacterial growth. Our impression is that NPWT enhances tissue enmeshing into GranuFoam™, and without intermittent removal higher bacterial loads may be seen. Lastly, this study reveals the potential role of a FBR to GranuFoam™ as a potential fundamental component to outcomes seen with NPWT. Insight into cell recruitment and genomic profiling for the different stages of wound healing could provide improved understanding of important signals that prompt the subsequent cascading series of events seen later.

## CONFLICT OF INTEREST

All authors declare that they do not have any conflict of interest except Dr David Zamierowski. Dr Zamierowski declares that he has sold patents to the VAC to KCI/Acelity and continues to receive royalties on Prevena (KCI/Acelity now known as KCI + 3M). Dr David Zamierowski is the owner and founder of Zam Research LLC.

## AUTHOR CONTRIBUTIONS

The study design was conceived by Ashley L. Pistorio, Richard A. Korentager, David S. Zamierowski and Adam J. Mellott. The study was executed by Ashley L. Pistorio, Jennifer G. Nelson‐Brantley, Molly E. Steed and Adam J. Mellott. Data analysis was performed by Jacob G. Hodge, Ashley L. Pistorio, Christopher A. Neal, Hongyan Dai and Molly E. Steed. Interpretation was performed by Jacob G. Hodge, Ashley L. Pistorio, Christopher A. Neal, Richard A. Korentager, David S. Zamierowski and Adam J. Mellott. The manuscript was written by Jacob G. Hodge, Ashley L. Pistorio, Christopher A. Neal, David S. Zamierowski and Adam J. Mellott.

## Supporting information


**Figure S1** Extracellular matrix enmeshing into GranuFoam™. Tissue samples explanted from pigs at day 3, 6 and 9 were histologically stained and analyzed under light microscopy. Regions near wound bed/edge were assessed to determine impact of tissue ingrowth/enmeshing from wound edges. Masson's Trichrome images at 200x magnification comparing non‐NPWT (*top row*) and NPWT (*bottom row*) over the time points of day 3 (*first column*), day 6 (*second column*), and day 9 (*third column*). Inset is image at 100x magnification. Masson's Trichrome images highlights collagen fibers (blue) and other matrix‐derived components (red). Scale Bar = 100 μm for 200x, and 200 μm for 100x (inset).

**Figure S2** Supplemental Picrosirius red and Alcian blue staining for matrix characterization of wound bed. Tissue samples explanted from pigs at day 9 were histologically stained and analyzed under light microscopy. Regions within GranuFoam™ and wound bed were assessed to characterize involvement of matrix‐derived compounds. Picrosirius Red under polarized light highlights collagen type I (*red/orange*) and collagen type III (*green*) and allows differentiation between the two. Alcian Blue stains sulfated glycosaminoglycans (GAGs) (*blue*) and cytoplasm (*pink*). Non‐NPWT (*left column set*) and NPWT (*right column set*) were examined at three different magnifications including 12.5x (*top row*), 40x (*middle row*) and 200x (*bottom row*). White box in 12.5x magnification indicate region of interest for 40x image, and white box in 40x indicates region of interest in 200x image. Leading edge of positive GAGs staining in Alcian Blue (*white arrow heads*) is shown in 12.5x images. Sulfated GAG encapsulation of GranuFoam™ particles (*white solid arrows*) is shown in 200x images. Collagen type III (*green*) and collagen type I (*red/orange*) encapsulation of GranuFoam™ (*dashed white arrows*) are highlighted in 200x images. Scale Bar = 1000 μm for 12.5x, 500 μm for 40x, and 100 μm for 200x.

**Figure S3** Early Changes in Wound Healing Gene Expression. A custom 1‐cm x 1‐cm x 1‐cm wound biopsy punch was used to inflict all wounds (day 0, 3, 6, and 9) and tissue was processed and analyzed as day 0 baseline tissue control samples. Subsequently, day 0 wounds were then elliptically explanted and analyzed as day 0 wounds, which occurred within the first 60 minutes of initial wounding. Both day 0 baseline tissue and day 0 wound tissue were analyzed for expression of key genes via a wound healing array. Values are reported as fold change against their respective gene expression to baseline tissue biopsies and normalized to a group of endogenous control genes, that included GAPDH, ACTB, HPRT1, and RPL13A. Comparison of day 0 wound tissue samples to day 0 baseline tissue samples indicated a number of significant genes within the first 60 minute timeframe of wounding to processing. A student's unpaired t‐test was used for statistical analysis. Significance denoted as *p < 0.05, **p < 0.01, and ***p < 0.001.

**Figure S4** Wound Healing Gene Expression – ECM Structural. Elliptically explanted wound tissue was assessed for expression of key genes via a wound healing array. Genes involved with the ECM Structural process of wound healing were grouped together and analyzed at days 3, 6, and 9 post‐injury, relative to baseline tissue controls. Values are reported as fold change against their respective gene expression to baseline tissue biopsies and normalized to a group of endogenous control genes, that included GAPDH, ACTB, HPRT1, and RPL13A. Each graph compares intragroup temporal differences and intraday difference between non‐NPWT and NPWT. Non‐NPWT (*left set*) and NPWT (*right set*) average fold changes are depicting temporally with day (*light grey*), day 6 (*dark grey*), and day 9 (*black*). A dashed line at a value of ‘1’ is used to depict average baseline expression. Error bars are s.e.m. and include n = 4. Significance on non‐NPWT and NPWT wounds relative to the baseline tissue is denoted with a ‘**#**’ above bar and indicates a p < 0.05. Intragroup and intraday significance is denoted as *p < 0.05, **p < 0.01, and ***p < 0.001.

**Figure S5** Wound Healing Gene Expression – ECM Remodeling. Elliptically explanted wound tissue was assessed for expression of key genes via a wound healing array. Genes involved with the ECM Remodeling process of wound healing were grouped together and analyzed at days 3, 6, and 9 post‐injury, relative to baseline tissue controls. Values are reported as fold change against their respective gene expression to baseline tissue biopsies and normalized to a group of endogenous control genes, that included GAPDH, ACTB, HPRT1, and RPL13A. Each graph compares intragroup temporal differences and intraday difference between non‐NPWT and NPWT. Non‐NPWT (*left set*) and NPWT (*right set*) average fold changes are depicting temporally with day (*light grey*), day 6 (*dark grey*), and day 9 (*black*). A dashed line at a value of ‘1’ is used to depict average baseline expression. Error bars are s.e.m. and include n = 4. Significance on non‐NPWT and NPWT wounds relative to the baseline tissue is denoted with a ‘**#**’ above bar and indicates a p < 0.05. Intragroup and intraday significance is denoted as *p < 0.05, **p < 0.01, and ***p < 0.001.

**Figure S6** Wound Healing Gene Expression – Cell Adhesion. Elliptically explanted wound tissue was assessed for expression of key genes via a wound healing array. Genes involved with the Cell Adhesion process of wound healing were grouped together and analyzed at days 3, 6, and 9 post‐injury, relative to baseline tissue controls. Values are reported as fold change against their respective gene expression to baseline tissue biopsies and normalized to a group of endogenous control genes, that included GAPDH, ACTB, HPRT1, and RPL13A. Each graph compares intragroup temporal differences and intraday difference between non‐NPWT and NPWT. Non‐NPWT (*left set*) and NPWT (*right set*) average fold changes are depicting temporally with day (*light grey*), day 6 (*dark grey*), and day 9 (*black*). A dashed line at a value of ‘1’ is used to depict average baseline expression. Error bars are s.e.m. and include n = 4. Significance on non‐NPWT and NPWT wounds relative to the baseline tissue is denoted with a ‘**#**’ above bar and indicates a p < 0.05. Intragroup and intraday significance is denoted as *p < 0.05, **p < 0.01, and ***p < 0.001.

**Figure S7** Wound Healing Gene Expression – Inflammation. Elliptically explanted wound tissue was assessed for expression of key genes via a wound healing array. Genes involved with the Inflammation process of wound healing were grouped together and analyzed at days 3, 6, and 9 post‐injury, relative to baseline tissue controls. Values are reported as fold change against their respective gene expression to baseline tissue biopsies and normalized to a group of endogenous control genes, that included GAPDH, ACTB, HPRT1, and RPL13A. Each graph compares intragroup temporal differences and intraday difference between non‐NPWT and NPWT. Non‐NPWT (*left set*) and NPWT (*right set*) average fold changes are depicting temporally with day (*light grey*), day 6 (*dark grey*), and day 9 (*black*). A dashed line at a value of ‘1’ is used to depict average baseline expression. Error bars are s.e.m. and include n = 4. Significance on non‐NPWT and NPWT wounds relative to the baseline tissue is denoted with a ‘**#**’ above bar and indicates a p < 0.05. Intragroup and intraday significance is denoted as *p < 0.05, **p < 0.01, and ***p < 0.001.

**Figure S8** Wound Healing Gene Expression – Growth Factors. Elliptically explanted wound tissue was assessed for expression of key genes via a wound healing array. Genes involved with the Growth Factors process of wound healing were grouped together and analyzed at days 3, 6, and 9 post‐injury, relative to baseline tissue controls. Values are reported as fold change against their respective gene expression to baseline tissue biopsies and normalized to a group of endogenous control genes, that included GAPDH, ACTB, HPRT1, and RPL13A. Each graph compares intragroup temporal differences and intraday difference between non‐NPWT and NPWT. Non‐NPWT (*left set*) and NPWT (*right set*) average fold changes are depicting temporally with day (*light grey*), day 6 (*dark grey*), and day 9 (*black*). A dashed line at a value of ‘1’ is used to depict average baseline expression. Error bars are s.e.m. and include n = 4. Significance on non‐NPWT and NPWT wounds relative to the baseline tissue is denoted with a ‘**#**’ above bar and indicates a p < 0.05. Intragroup and intraday significance is denoted as *p < 0.05, **p < 0.01, and ***p < 0.001.

**Figure S9** Wound Healing Gene Expression – Signal Transduction. Elliptically explanted wound tissue was assessed for expression of key genes via a wound healing array. Genes involved with the Signal Transduction process of wound healing were grouped together and analyzed at days 3, 6, and 9 post‐injury, relative to baseline tissue controls. Values are reported as fold change against their respective gene expression to baseline tissue biopsies and normalized to a group of endogenous control genes, that included GAPDH, ACTB, HPRT1, and RPL13A. Each graph compares intragroup temporal differences and intraday difference between non‐NPWT and NPWT. Non‐NPWT (*left set*) and NPWT (*right set*) average fold changes are depicting temporally with day (*light grey*), day 6 (*dark grey*), and day 9 (*black*). A dashed line at a value of ‘1’ is used to depict average baseline expression. Error bars are s.e.m. and include n = 4. Significance on non‐NPWT and NPWT wounds relative to the baseline tissue is denoted with a ‘**#**’ above bar and indicates a p < 0.05. Intragroup and intraday significance is denoted as *p < 0.05, **p < 0.01, and ***p < 0.001.

**Figure S10** Features of Foreign Body Response and Chronic Inflammation. Tissue samples explanted from pigs and day 9 samples are shown. Samples were histologically stained with H&E and analyzed under light microscopy. Regions Within the GranuFoam™ (*top row*) and Within the Wound tissue (*bottom row*) were assessed for presence Giant Cell formation (*left column*), Neovascularization (*middle column*), and Eosinophil Infiltration (*right column*). Giant Cells are highlighted with “white arrows” in all image sets, Neovascularization is highlights with “white circle” in image sets, and aggregates of Eosinophils are highlighted with “black arrows” in image sets. GranuFoam™ is denoted with “*”. Scale bar = 50 μm for all images.

**Table S1** List of Wound Healing Genes Included in RT‐PCR Array.

## Data Availability

The authors collectively declare that all data supporting the findings of the presented work are available within the paper and its supplementary information files.
