# Supplementary figures and images for: Novel insights into negative pressure wound healing from an in situ porcine perspective
Source: Wound Repair Regen. 2021 Oct 7;30(1):64–81. doi: 10.1111/wrr.12971 (PMC8724420; doi:10.1111/wrr.12971)

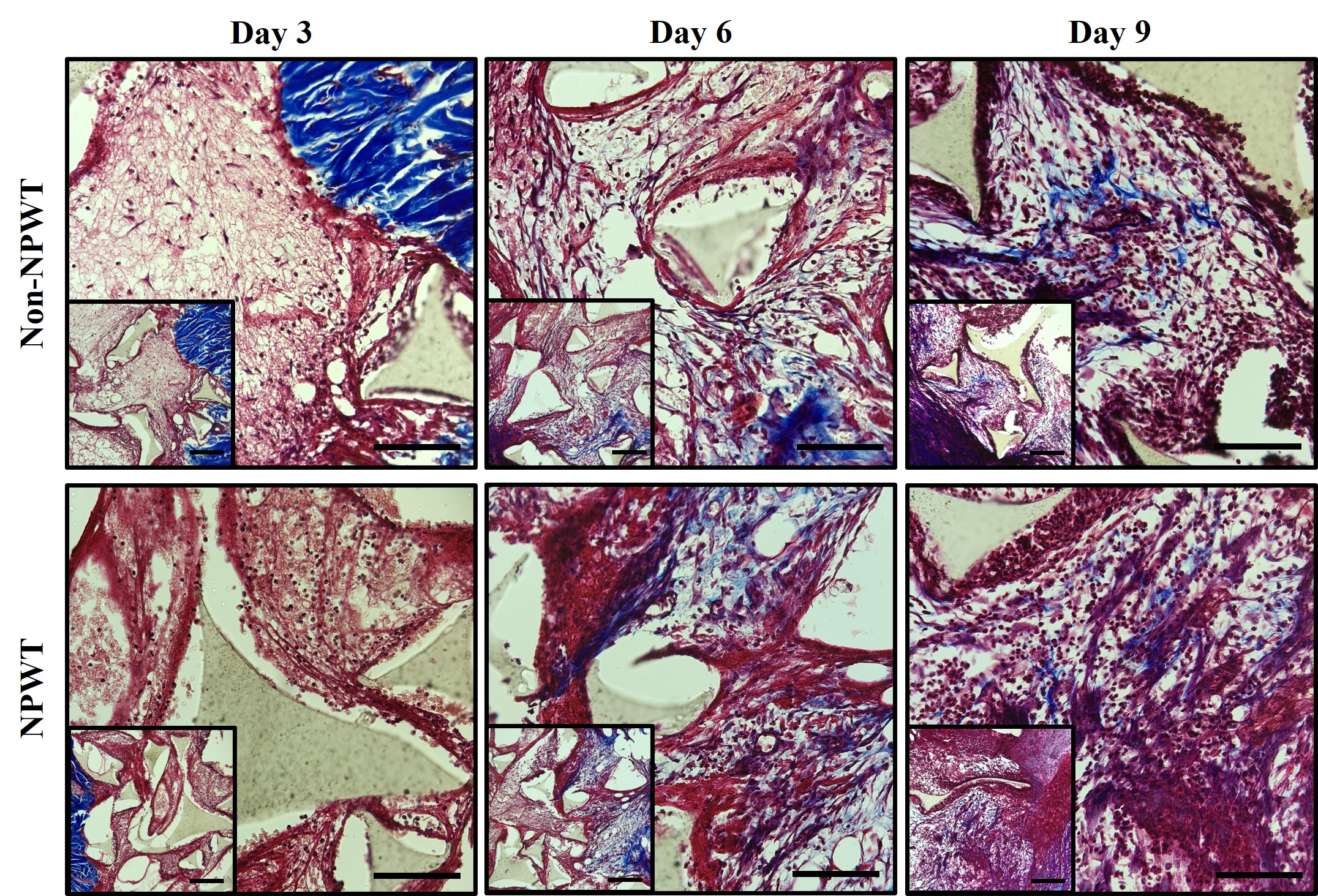

Supplement: Supplementary file 1 — Figure S1 Extracellular matrix enmeshing into GranuFoam™. Tissue samples explanted from pigs at day 3, 6 and 9 were histologically stained and analyzed under light microscopy. Regions near wound bed/edge were assessed to determine impact of tissue ingrowth/enmeshing from wound edges. Masson's Trichrome images at 200x magnification comparing non‐NPWT (top row) and NPWT (bottom row) over the time points of day 3 (first column), day 6 (second column), and day 9 (third column). Inset is image at 100x magnification. Masson's Trichrome images highlights collagen fibers (blue) and other matrix‐derived components (red). Scale Bar = 100 μm for 200x, and 200 μm for 100x (inset). [file WRR-30-64-s003.jpg]

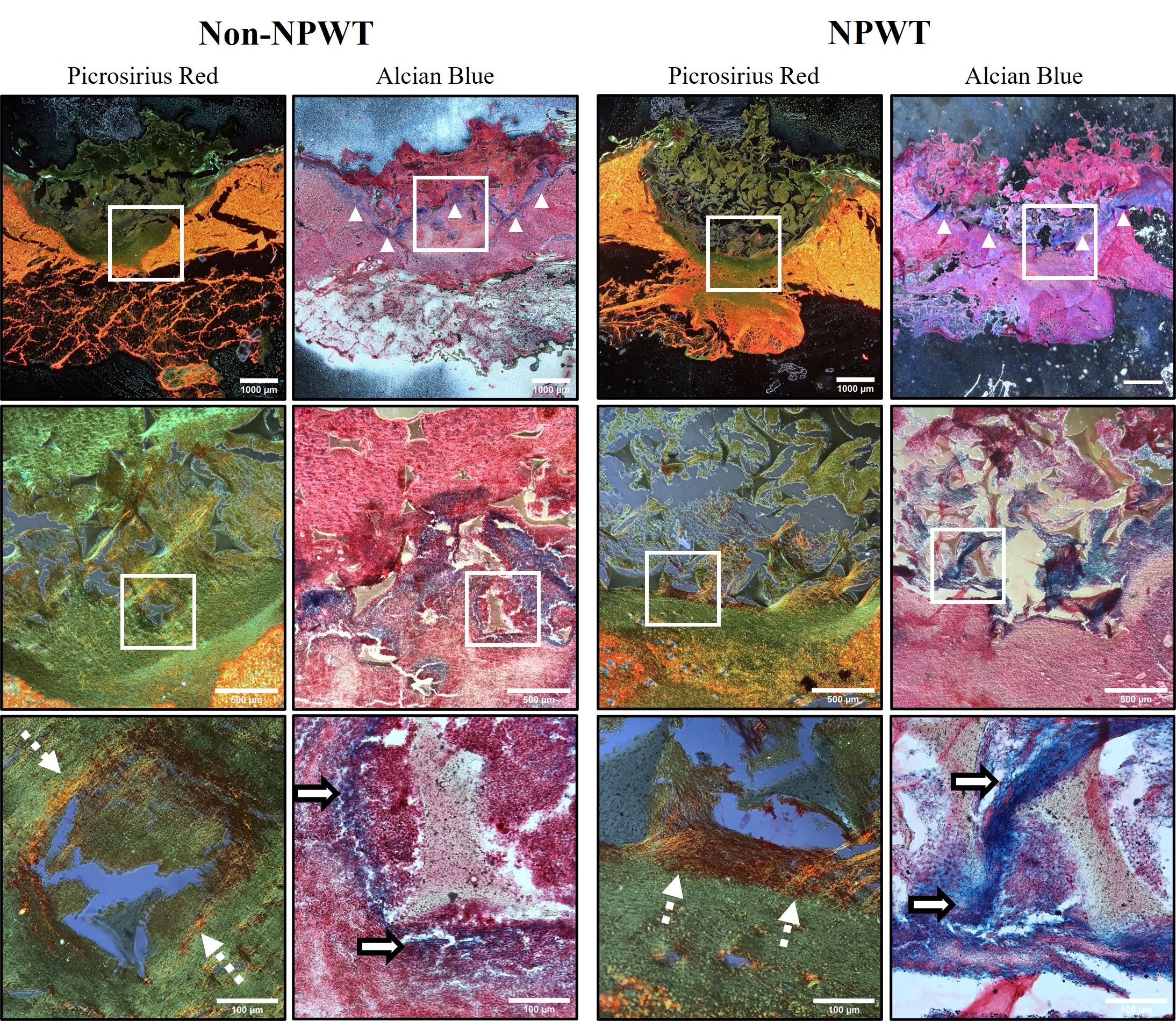

Supplement: Supplementary file 2 — Figure S2 Supplemental Picrosirius red and Alcian blue staining for matrix characterization of wound bed. Tissue samples explanted from pigs at day 9 were histologically stained and analyzed under light microscopy. Regions within GranuFoam™ and wound bed were assessed to characterize involvement of matrix‐derived compounds. Picrosirius Red under polarized light highlights collagen type I (red/orange) and collagen type III (green) and allows differentiation between the two. Alcian Blue stains sulfated glycosaminoglycans (GAGs) (blue) and cytoplasm (pink). Non‐NPWT (left column set) and NPWT (right column set) were examined at three different magnifications including 12.5x (top row), 40x (middle row) and 200x (bottom row). White box in 12.5x magnification indicate region of interest for 40x image, and white box in 40x indicates region of interest in 200x image. Leading edge of positive GAGs staining in Alcian Blue (white arrow heads) is shown in 12.5x images. Sulfated GAG encapsulation of GranuFoam™ particles (white solid arrows) is shown in 200x images. Collagen type III (green) and collagen type I (red/orange) encapsulation of GranuFoam™ (dashed white arrows) are highlighted in 200x images. Scale Bar = 1000 μm for 12.5x, 500 μm for 40x, and 100 μm for 200x. [file WRR-30-64-s002.jpg]

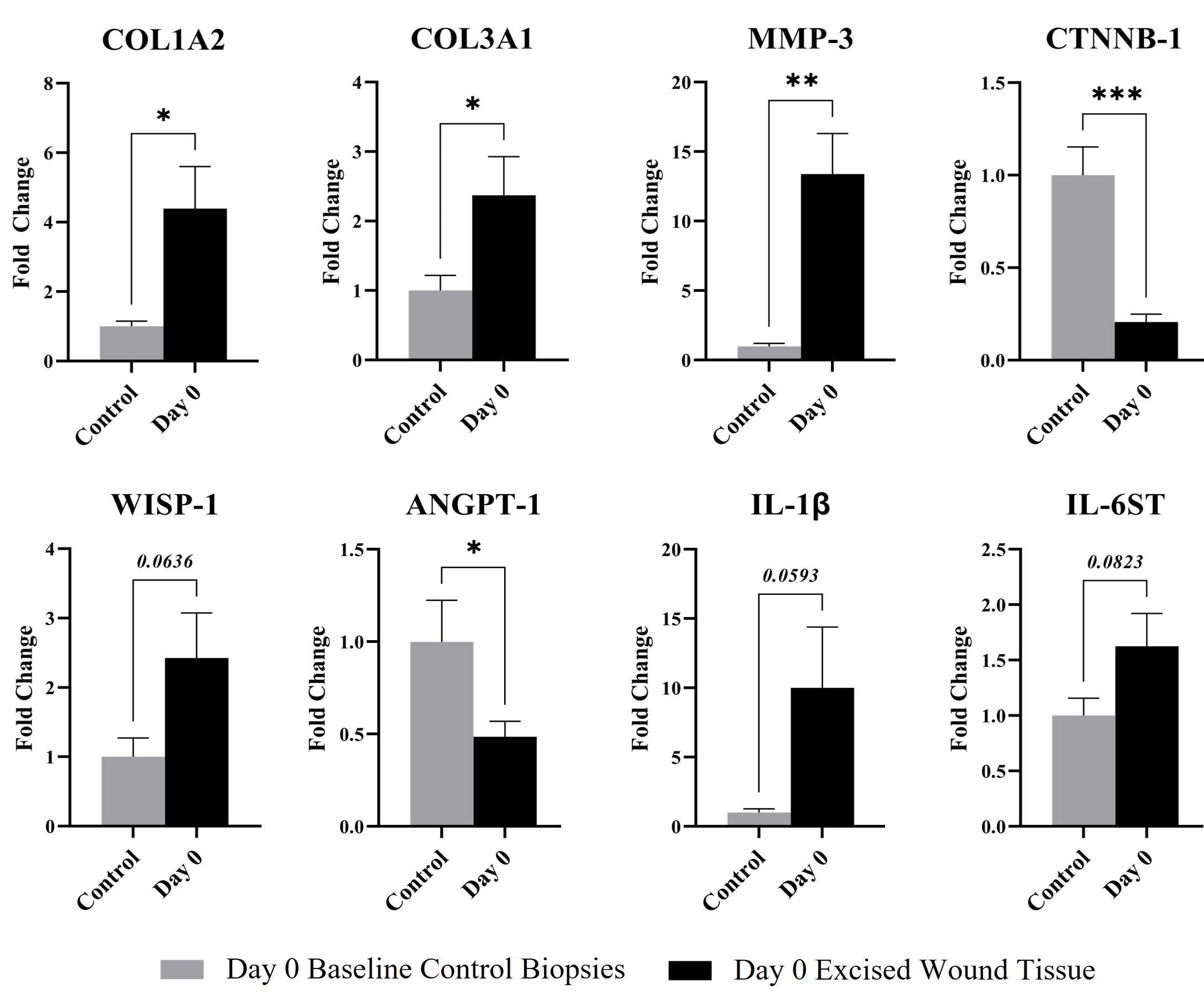

Supplement: Supplementary file 3 — Figure S3 Early Changes in Wound Healing Gene Expression. A custom 1‐cm x 1‐cm x 1‐cm wound biopsy punch was used to inflict all wounds (day 0, 3, 6, and 9) and tissue was processed and analyzed as day 0 baseline tissue control samples. Subsequently, day 0 wounds were then elliptically explanted and analyzed as day 0 wounds, which occurred within the first 60 minutes of initial wounding. Both day 0 baseline tissue and day 0 wound tissue were analyzed for expression of key genes via a wound healing array. Values are reported as fold change against their respective gene expression to baseline tissue biopsies and normalized to a group of endogenous control genes, that included GAPDH, ACTB, HPRT1, and RPL13A. Comparison of day 0 wound tissue samples to day 0 baseline tissue samples indicated a number of significant genes within the first 60 minute timeframe of wounding to processing. A student's unpaired t‐test was used for statistical analysis. Significance denoted as *p < 0.05, **p < 0.01, and ***p < 0.001. [file WRR-30-64-s008.jpg]

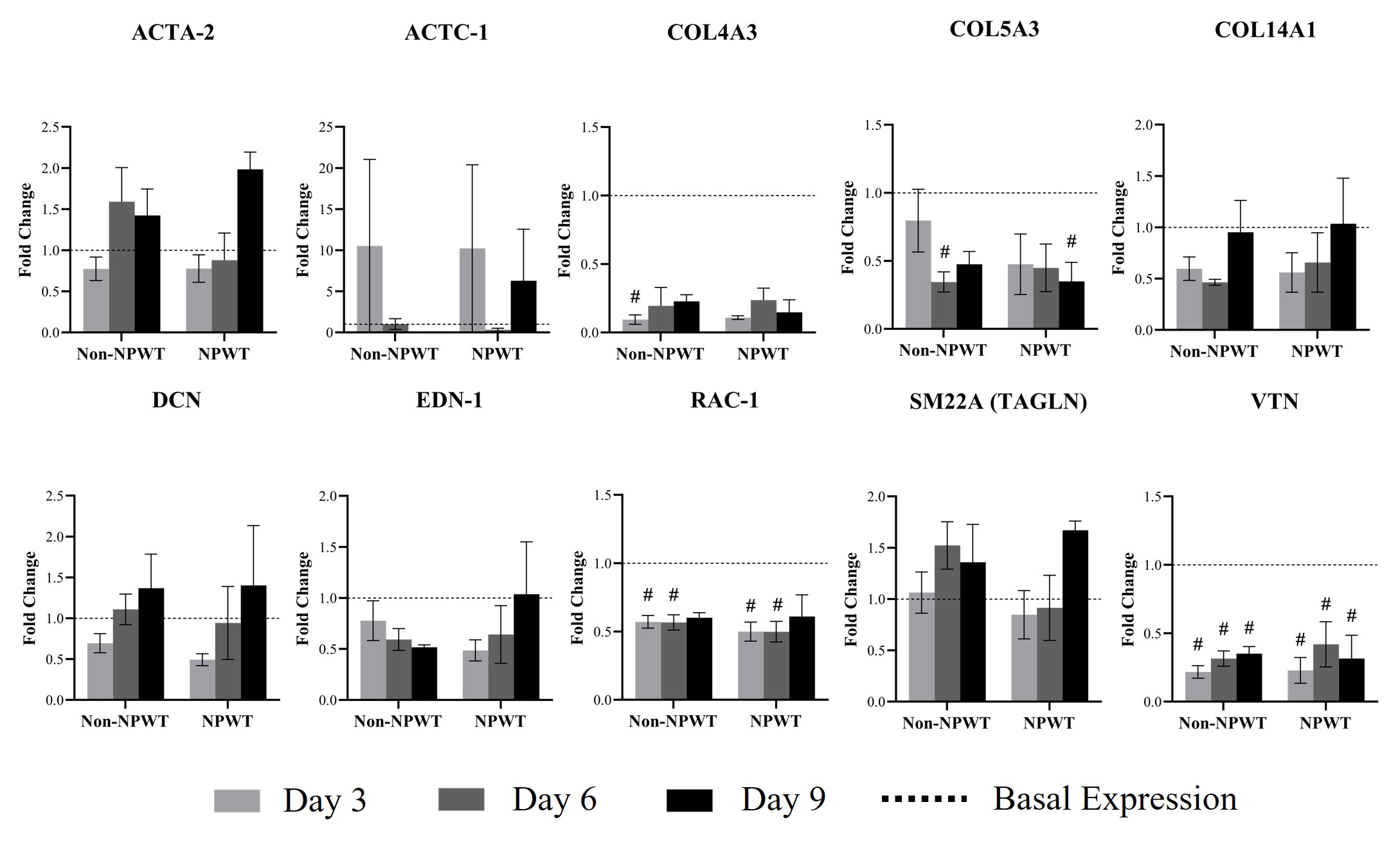

Supplement: Supplementary file 4 — Figure S4 Wound Healing Gene Expression – ECM Structural. Elliptically explanted wound tissue was assessed for expression of key genes via a wound healing array. Genes involved with the ECM Structural process of wound healing were grouped together and analyzed at days 3, 6, and 9 post‐injury, relative to baseline tissue controls. Values are reported as fold change against their respective gene expression to baseline tissue biopsies and normalized to a group of endogenous control genes, that included GAPDH, ACTB, HPRT1, and RPL13A. Each graph compares intragroup temporal differences and intraday difference between non‐NPWT and NPWT. Non‐NPWT (left set) and NPWT (right set) average fold changes are depicting temporally with day (light grey), day 6 (dark grey), and day 9 (black). A dashed line at a value of ‘1’ is used to depict average baseline expression. Error bars are s.e.m. and include n = 4. Significance on non‐NPWT and NPWT wounds relative to the baseline tissue is denoted with a ‘#’ above bar and indicates a p < 0.05. Intragroup and intraday significance is denoted as *p < 0.05, **p < 0.01, and ***p < 0.001. [file WRR-30-64-s001.jpg]

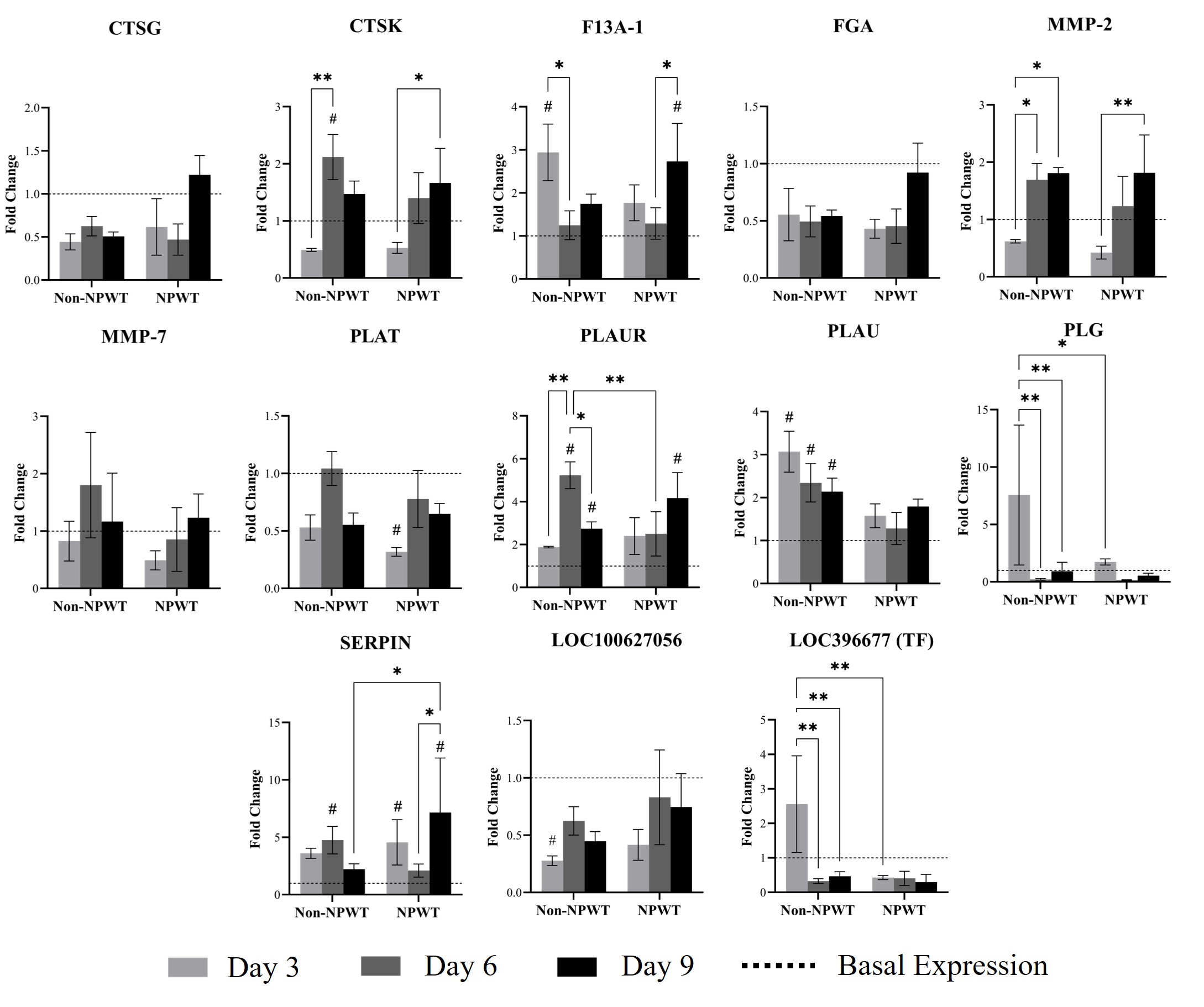

Supplement: Supplementary file 5 — Figure S5 Wound Healing Gene Expression – ECM Remodeling. Elliptically explanted wound tissue was assessed for expression of key genes via a wound healing array. Genes involved with the ECM Remodeling process of wound healing were grouped together and analyzed at days 3, 6, and 9 post‐injury, relative to baseline tissue controls. Values are reported as fold change against their respective gene expression to baseline tissue biopsies and normalized to a group of endogenous control genes, that included GAPDH, ACTB, HPRT1, and RPL13A. Each graph compares intragroup temporal differences and intraday difference between non‐NPWT and NPWT. Non‐NPWT (left set) and NPWT (right set) average fold changes are depicting temporally with day (light grey), day 6 (dark grey), and day 9 (black). A dashed line at a value of ‘1’ is used to depict average baseline expression. Error bars are s.e.m. and include n = 4. Significance on non‐NPWT and NPWT wounds relative to the baseline tissue is denoted with a ‘#’ above bar and indicates a p < 0.05. Intragroup and intraday significance is denoted as *p < 0.05, **p < 0.01, and ***p < 0.001. [file WRR-30-64-s011.jpg]

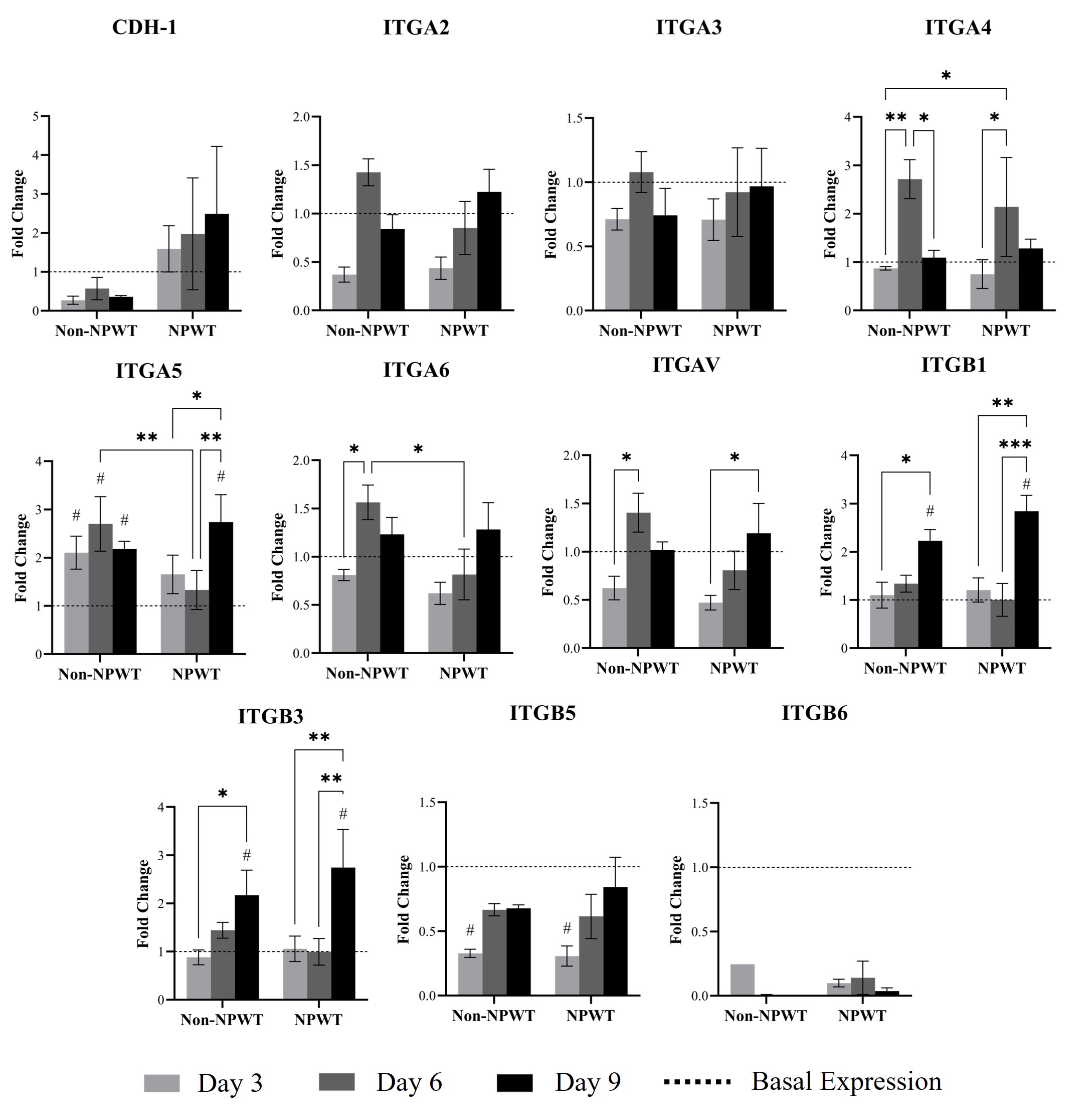

Supplement: Supplementary file 6 — Figure S6 Wound Healing Gene Expression – Cell Adhesion. Elliptically explanted wound tissue was assessed for expression of key genes via a wound healing array. Genes involved with the Cell Adhesion process of wound healing were grouped together and analyzed at days 3, 6, and 9 post‐injury, relative to baseline tissue controls. Values are reported as fold change against their respective gene expression to baseline tissue biopsies and normalized to a group of endogenous control genes, that included GAPDH, ACTB, HPRT1, and RPL13A. Each graph compares intragroup temporal differences and intraday difference between non‐NPWT and NPWT. Non‐NPWT (left set) and NPWT (right set) average fold changes are depicting temporally with day (light grey), day 6 (dark grey), and day 9 (black). A dashed line at a value of ‘1’ is used to depict average baseline expression. Error bars are s.e.m. and include n = 4. Significance on non‐NPWT and NPWT wounds relative to the baseline tissue is denoted with a ‘#’ above bar and indicates a p < 0.05. Intragroup and intraday significance is denoted as *p < 0.05, **p < 0.01, and ***p < 0.001. [file WRR-30-64-s010.jpg]

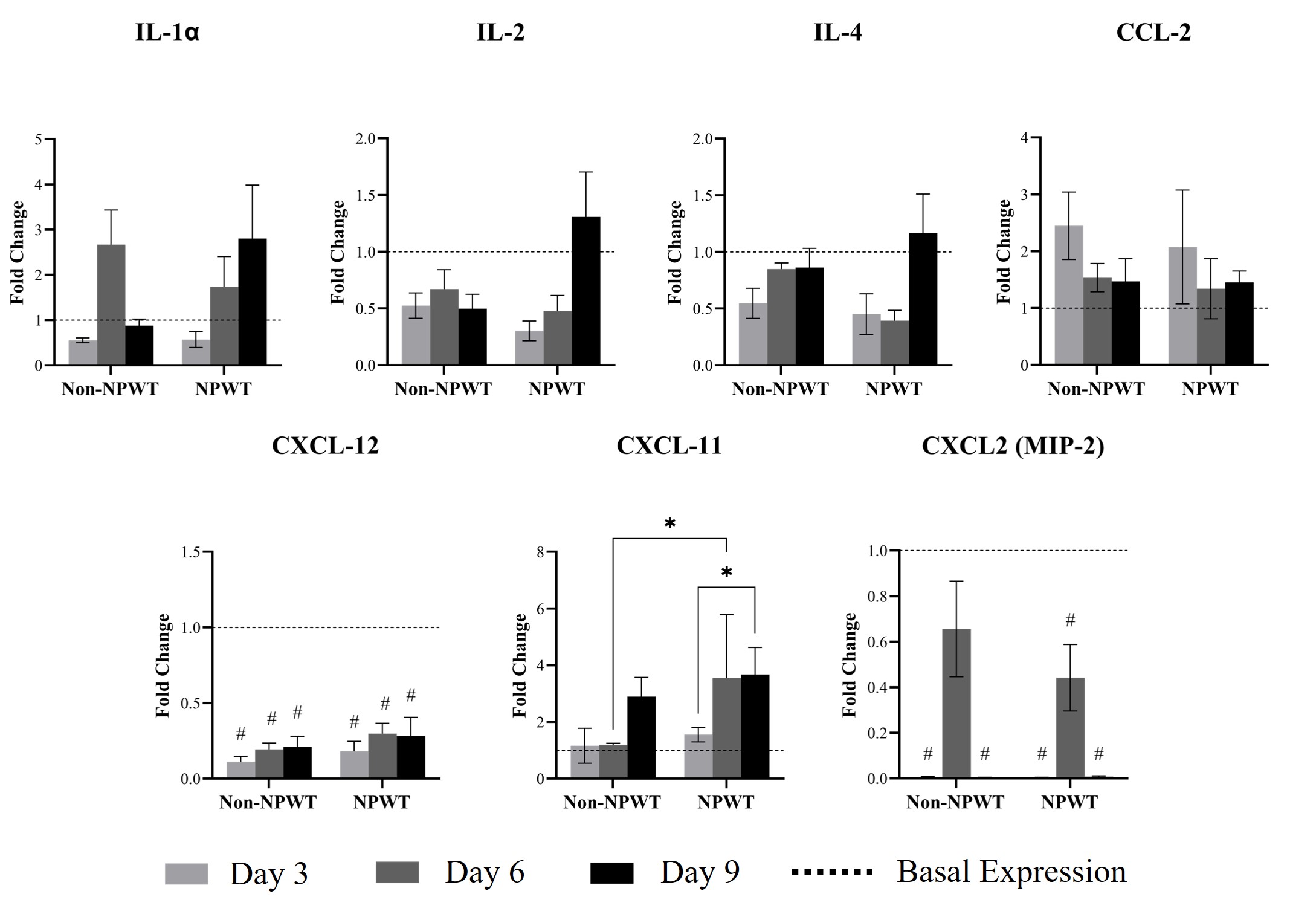

Supplement: Supplementary file 7 — Figure S7 Wound Healing Gene Expression – Inflammation. Elliptically explanted wound tissue was assessed for expression of key genes via a wound healing array. Genes involved with the Inflammation process of wound healing were grouped together and analyzed at days 3, 6, and 9 post‐injury, relative to baseline tissue controls. Values are reported as fold change against their respective gene expression to baseline tissue biopsies and normalized to a group of endogenous control genes, that included GAPDH, ACTB, HPRT1, and RPL13A. Each graph compares intragroup temporal differences and intraday difference between non‐NPWT and NPWT. Non‐NPWT (left set) and NPWT (right set) average fold changes are depicting temporally with day (light grey), day 6 (dark grey), and day 9 (black). A dashed line at a value of ‘1’ is used to depict average baseline expression. Error bars are s.e.m. and include n = 4. Significance on non‐NPWT and NPWT wounds relative to the baseline tissue is denoted with a ‘#’ above bar and indicates a p < 0.05. Intragroup and intraday significance is denoted as *p < 0.05, **p < 0.01, and ***p < 0.001. [file WRR-30-64-s005.jpg]

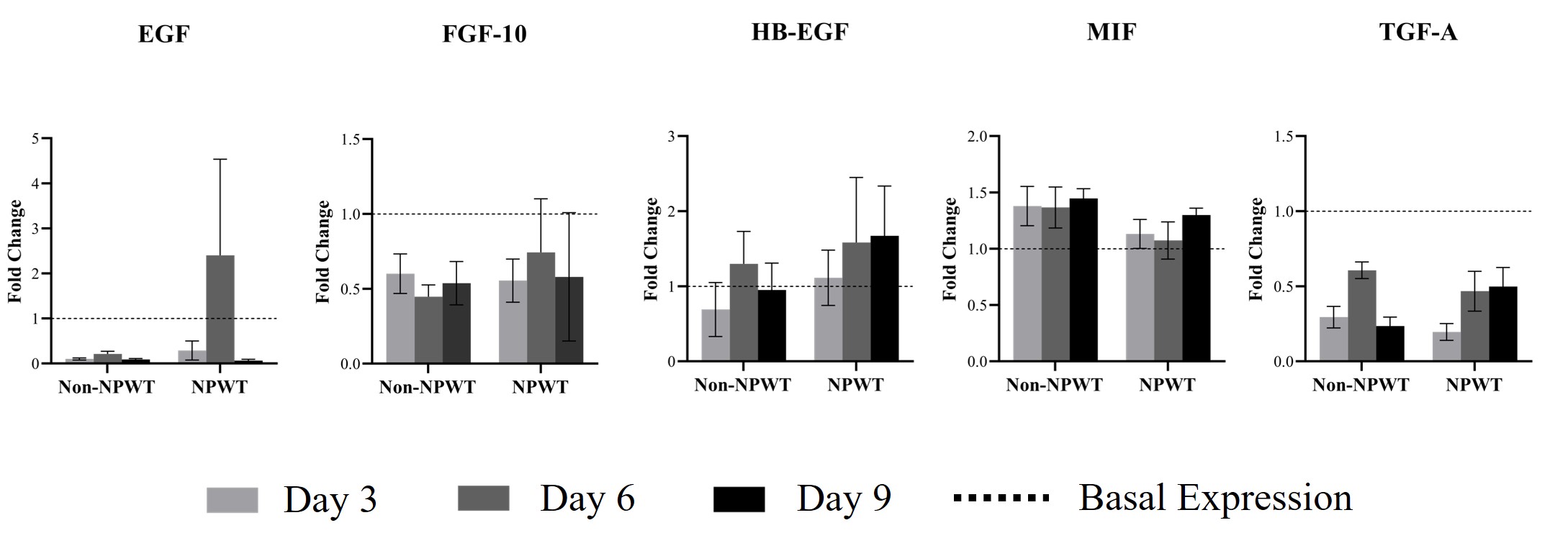

Supplement: Supplementary file 8 — Figure S8 Wound Healing Gene Expression – Growth Factors. Elliptically explanted wound tissue was assessed for expression of key genes via a wound healing array. Genes involved with the Growth Factors process of wound healing were grouped together and analyzed at days 3, 6, and 9 post‐injury, relative to baseline tissue controls. Values are reported as fold change against their respective gene expression to baseline tissue biopsies and normalized to a group of endogenous control genes, that included GAPDH, ACTB, HPRT1, and RPL13A. Each graph compares intragroup temporal differences and intraday difference between non‐NPWT and NPWT. Non‐NPWT (left set) and NPWT (right set) average fold changes are depicting temporally with day (light grey), day 6 (dark grey), and day 9 (black). A dashed line at a value of ‘1’ is used to depict average baseline expression. Error bars are s.e.m. and include n = 4. Significance on non‐NPWT and NPWT wounds relative to the baseline tissue is denoted with a ‘#’ above bar and indicates a p < 0.05. Intragroup and intraday significance is denoted as *p < 0.05, **p < 0.01, and ***p < 0.001. [file WRR-30-64-s006.jpg]

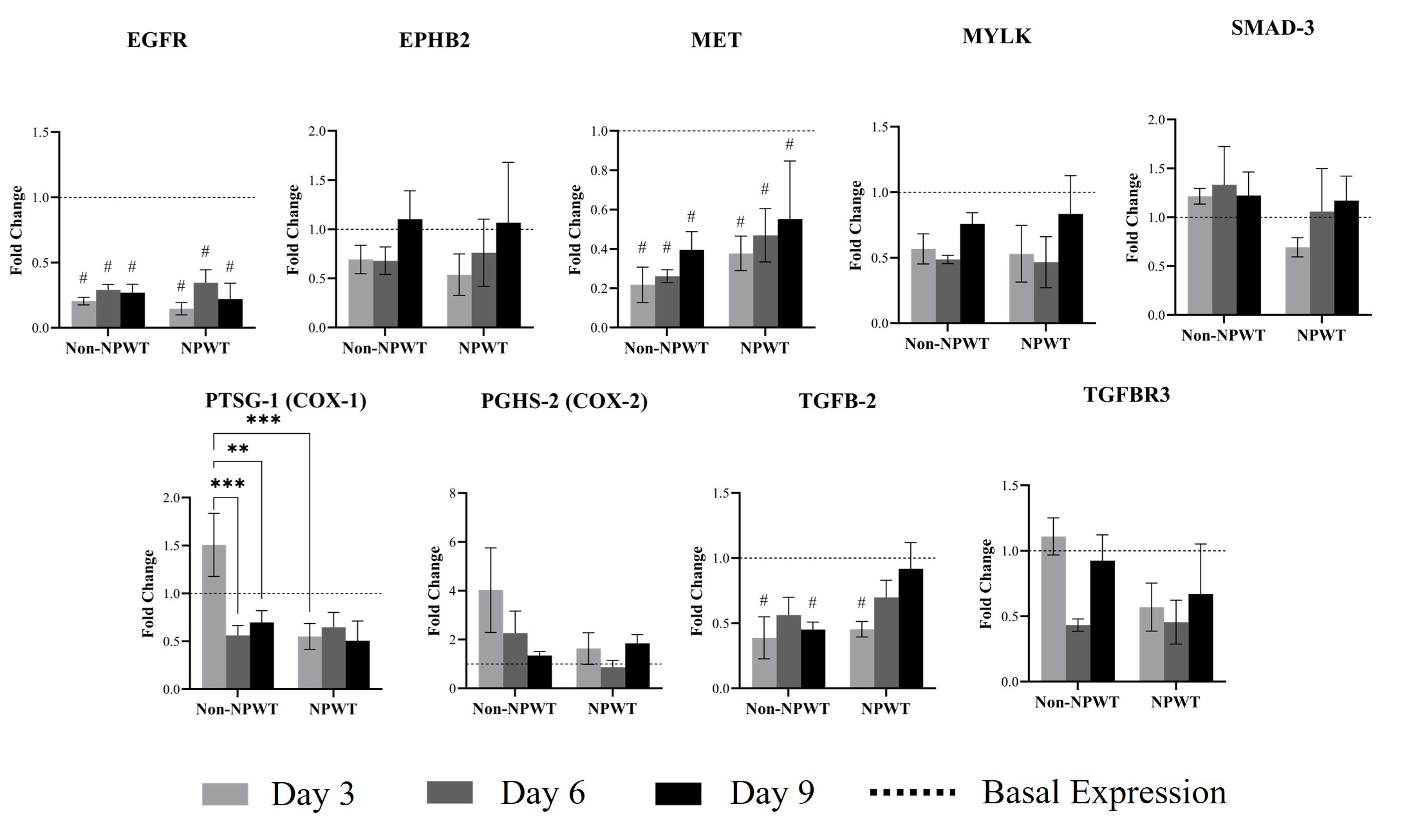

Supplement: Supplementary file 9 — Figure S9 Wound Healing Gene Expression – Signal Transduction. Elliptically explanted wound tissue was assessed for expression of key genes via a wound healing array. Genes involved with the Signal Transduction process of wound healing were grouped together and analyzed at days 3, 6, and 9 post‐injury, relative to baseline tissue controls. Values are reported as fold change against their respective gene expression to baseline tissue biopsies and normalized to a group of endogenous control genes, that included GAPDH, ACTB, HPRT1, and RPL13A. Each graph compares intragroup temporal differences and intraday difference between non‐NPWT and NPWT. Non‐NPWT (left set) and NPWT (right set) average fold changes are depicting temporally with day (light grey), day 6 (dark grey), and day 9 (black). A dashed line at a value of ‘1’ is used to depict average baseline expression. Error bars are s.e.m. and include n = 4. Significance on non‐NPWT and NPWT wounds relative to the baseline tissue is denoted with a ‘#’ above bar and indicates a p < 0.05. Intragroup and intraday significance is denoted as *p < 0.05, **p < 0.01, and ***p < 0.001. [file WRR-30-64-s004.jpg]

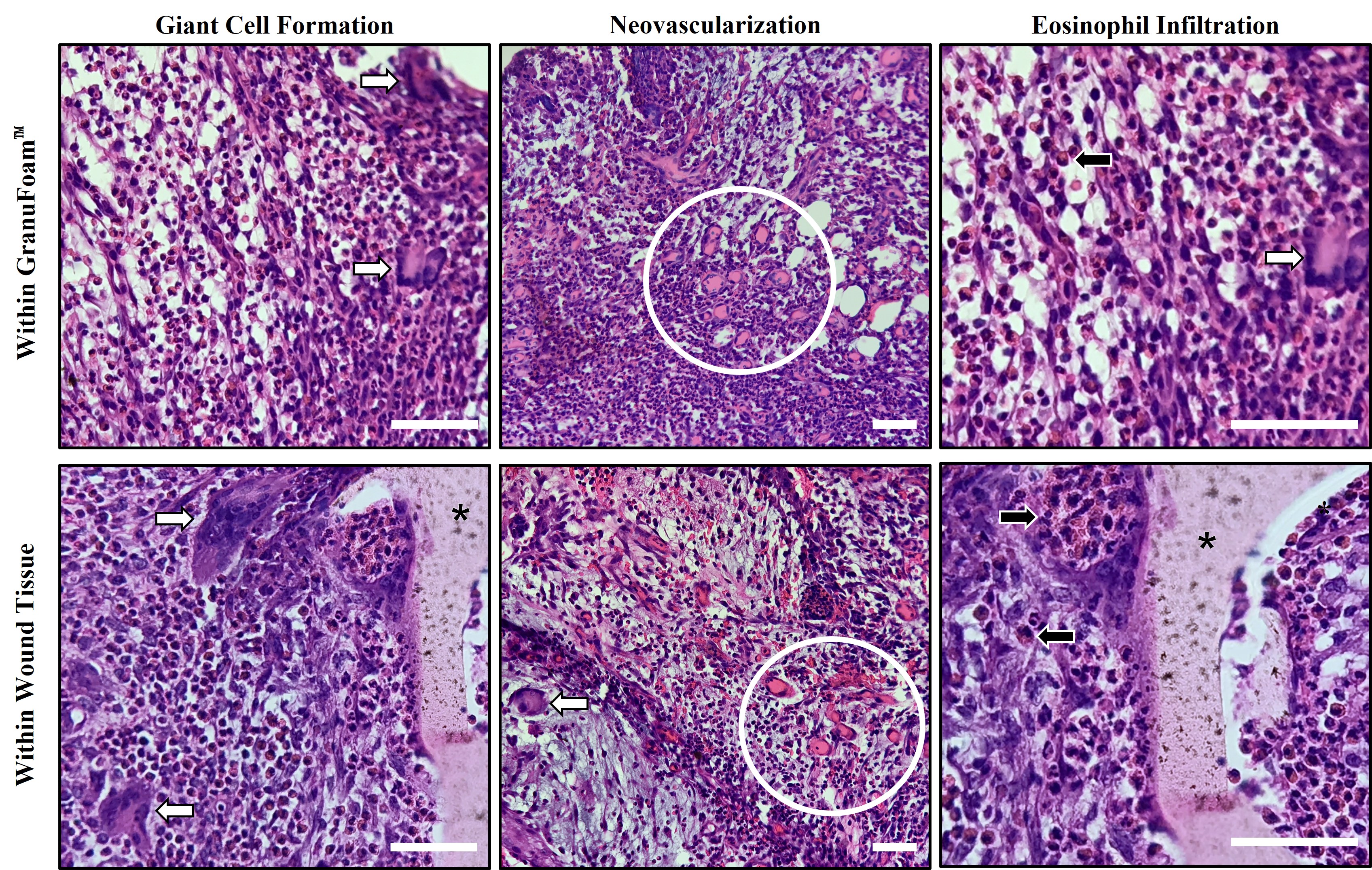

Supplement: Supplementary file 10 — Figure S10 Features of Foreign Body Response and Chronic Inflammation. Tissue samples explanted from pigs and day 9 samples are shown. Samples were histologically stained with H&E and analyzed under light microscopy. Regions Within the GranuFoam™ (top row) and Within the Wound tissue (bottom row) were assessed for presence Giant Cell formation (left column), Neovascularization (middle column), and Eosinophil Infiltration (right column). Giant Cells are highlighted with “white arrows” in all image sets, Neovascularization is highlights with “white circle” in image sets, and aggregates of Eosinophils are highlighted with “black arrows” in image sets. GranuFoam™ is denoted with “*”. Scale bar = 50 μm for all images. [file WRR-30-64-s009.jpg]

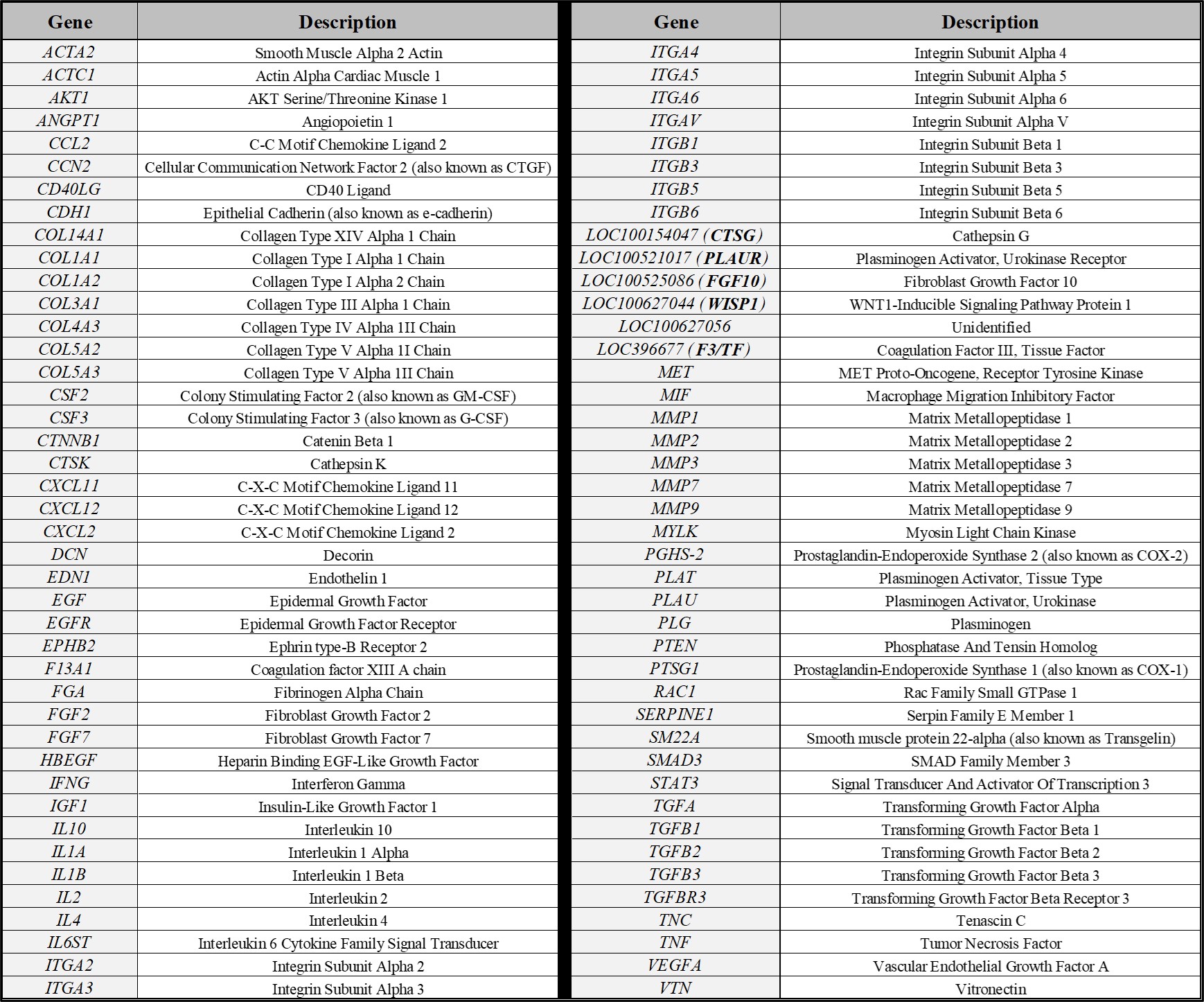

Supplement: Supplementary file 11 — Table S1 List of Wound Healing Genes Included in RT‐PCR Array. [file WRR-30-64-s007.jpg]
